# Supplementary material for: Berry curvature-induced local spin polarisation in gated graphene/WTe2 heterostructures
Source: Nat Commun. 2022 Jun 7;13:3152. doi: 10.1038/s41467-022-30744-3 (PMC9174237; doi:10.1038/s41467-022-30744-3)
Supplement: Supplementary file 1 — Supplementary Information [file 41467_2022_30744_MOESM1_ESM.pdf]

Supplementary Information for "Berry curvature-induced local spin polarisation in gated graphene/WTe_2 heterostructures"

Lukas Powalla[1,2]*, Jonas Kiemle[3,4]*, Elio J. König[1], Andreas P. Schnyder[1], Johannes Knolle[4,5,6], Klaus Kern[1,2], Alexander Holleitner[3,4], Christoph Kastl[3,4] and Marko Burghard[1]

1 Max-Planck-Institut für Festkörperforschung, Heisenbergstrasse 1, D-70569 Stuttgart, Germany.
2 Institut de Physique, Ecole Polytechnique Fédérale de Lausanne, CH-1015 Lausanne, Switzerland.
3 Walter Schottky Institut and Physics Department,
Technical University of Munich, Am Coulombwall 4a, D-85748 Garching, Germany.
4 MCQST, Schellingstrasse 4, D-80799 München, Germany.
5 Department of Physics TQM, Technical University of Munich,
James-Frank-Strasse 1, D-85748 Garching, Germany.
6 Faculty of Natural Sciences, Department of Physics,
Imperial College London, London SW7 2AZ, UK.
** these authors contributed equally to this work.*

Raman spectroscopy

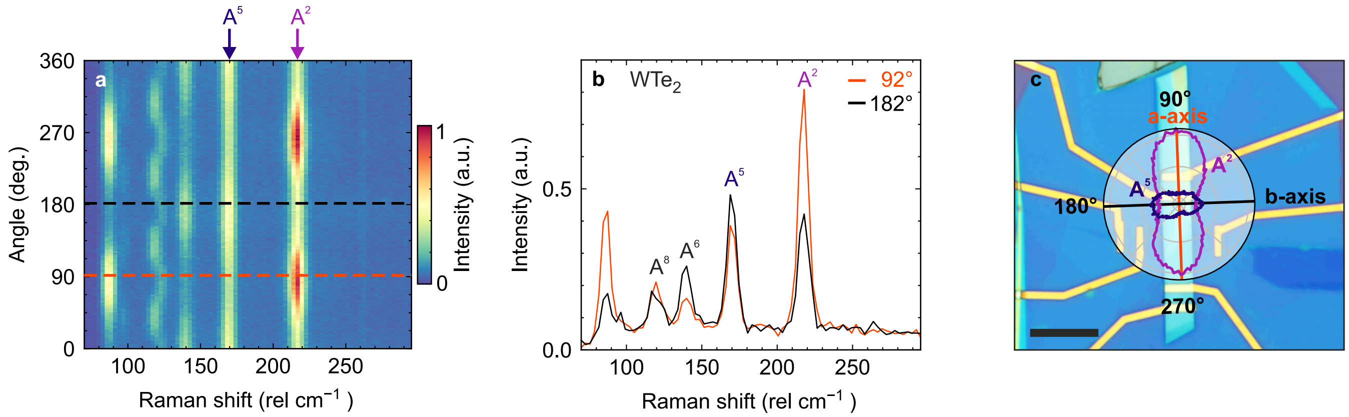

Supplementary Fig. S1. **Raman spectroscopy.** a) Polarisation-resolved Raman spectra of the WTe_2 ribbon in Fig. 1 of the main manuscript for different linear excitation polarisations at room temperature. The linearly polarised laser excitation ($\lambda_{exc} = 532\,\text{nm}$) is rotated with a half-waveplate. The signal is detected in co-polarisation configuration, i.e. excitation polariser and detection analyzer are parallel to each other. b) Raman spectra of the WTe_2 film along its crystallographic *a*- (orange line) and *b*-axis (black line) colour coded in panels a) (dashed lines) and c) (vertical and horizontal lines), respectively. c) Optical image of the graphene/WTe_2 cross-junction covered with hBN. A polarisation-dependent intensity polar plot of the A^2- (purple) and A^5-Raman modes (blue) is overlaid, confirming the crystallographic axis orientations. Scale bar is 10 µm.

KR microscopy - gate and bias dependence

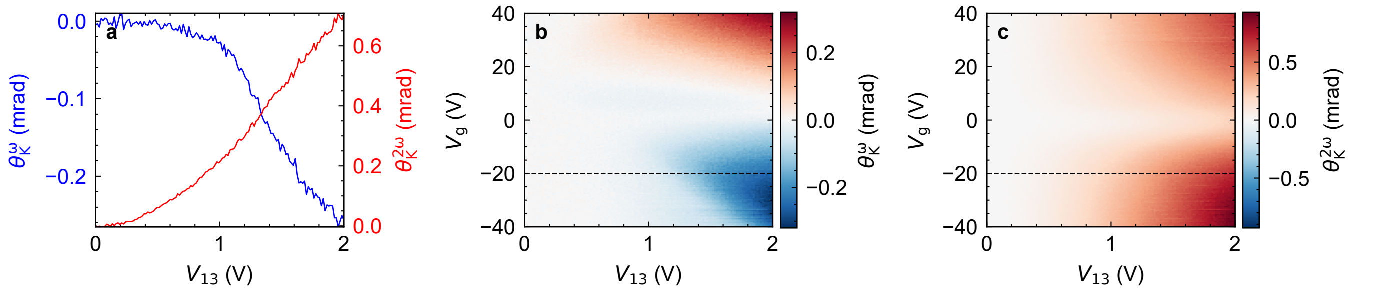

Supplementary Fig. S2. **Bias- and gate-dependent KR microscopy.** a) First (blue line) and second harmonic (red line) KR signal as a function of ac bias along the graphene stripe (contacts labelled 1 and 3 in Fig. 1 of the main manuscript), acquired on the junction edge at $V_\mathrm{g} = -20\,\mathrm{V}$. While the 2ω KR scales approximately quadratic with the applied ac bias, the KR at the fundamental frequency ω exhibits a gate voltage-dependent onset. b)-c) First and second harmonic KR angle as a function of applied graphene ac bias and gate voltage. The dashed lines indicate the traces depicted in panel a).

Data of additional samples

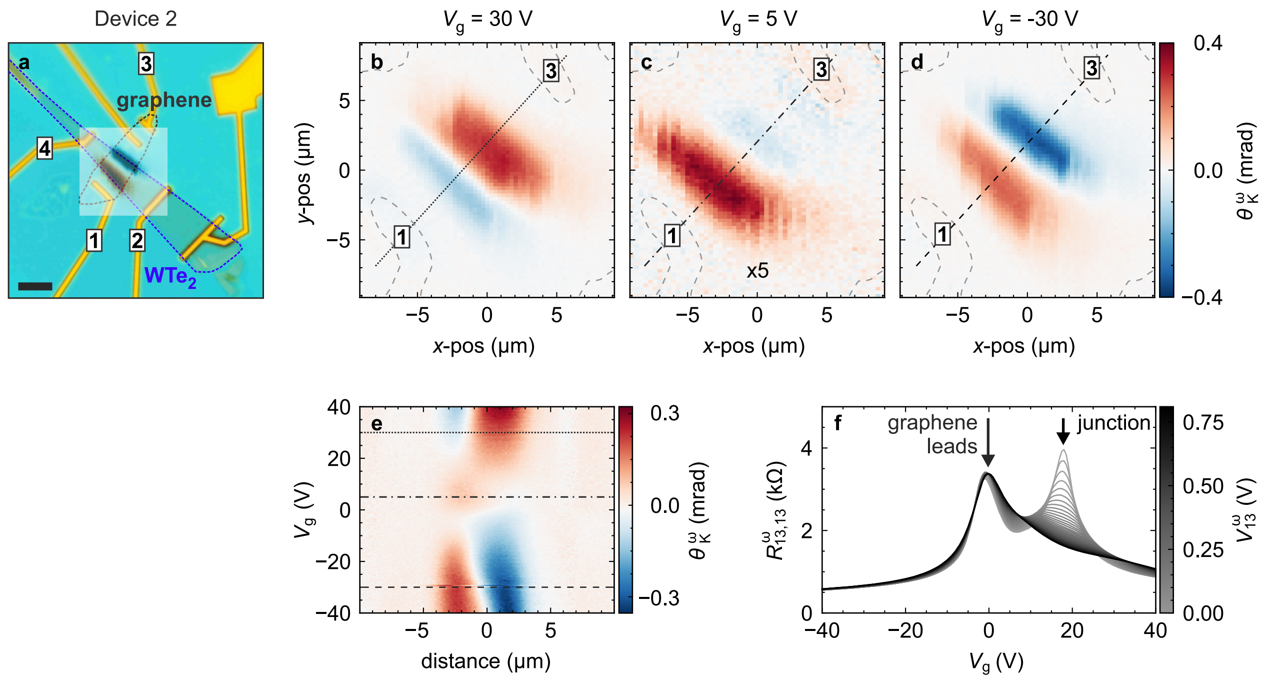

Supplementary Fig. S3. **Extended data of a second device.** a) Optical microscopy image of a heterostructure comprised of graphene (black dashed line), WTe_2 (blue dashed line), and hBN capping. Scale bar is 5 µm. The overlay (shaded area) shows the current-induced KR signal at the junction using a colour code as in panel (d), for a current applied between the contacts labelled 1 and 3. b)-d) Spatially-resolved magneto-optic KR signal under ac current flow along the graphene stripe for (b) $V_\mathrm{g} = 30\,\mathrm{V}$, (c) $V_\mathrm{g} = 5\,\mathrm{V}$, (d) $V_\mathrm{g} = -30\,\mathrm{V}$. The Kerr angles are measured at the fundamental frequency ω of the alternating bias current. Grey dashed lines highlight the metal electrodes. The data in c) is scaled by a factor of 5 for clarity. $T_\mathrm{bath} = 4.2\,\mathrm{K}$, $V_{13}^{\omega} = 2\,\mathrm{V}$. e) Spatially-resolved KR θ_K^{ω} across the graphene/WTe_2 heterojunction as a function of applied gate voltage. Dotted, dash-dotted and dashed lines correspond to profiles indicated in panels b), c), and d), respectively. f) Two-terminal resistance $R_{13,13}^{\omega}$ of the graphene stripe measured between contacts 1 and 3 of the device in panel (a), at $T_\mathrm{bath} = 4.2\,\mathrm{K}$ using an ac voltage that increases stepwise up to 0.8 V.

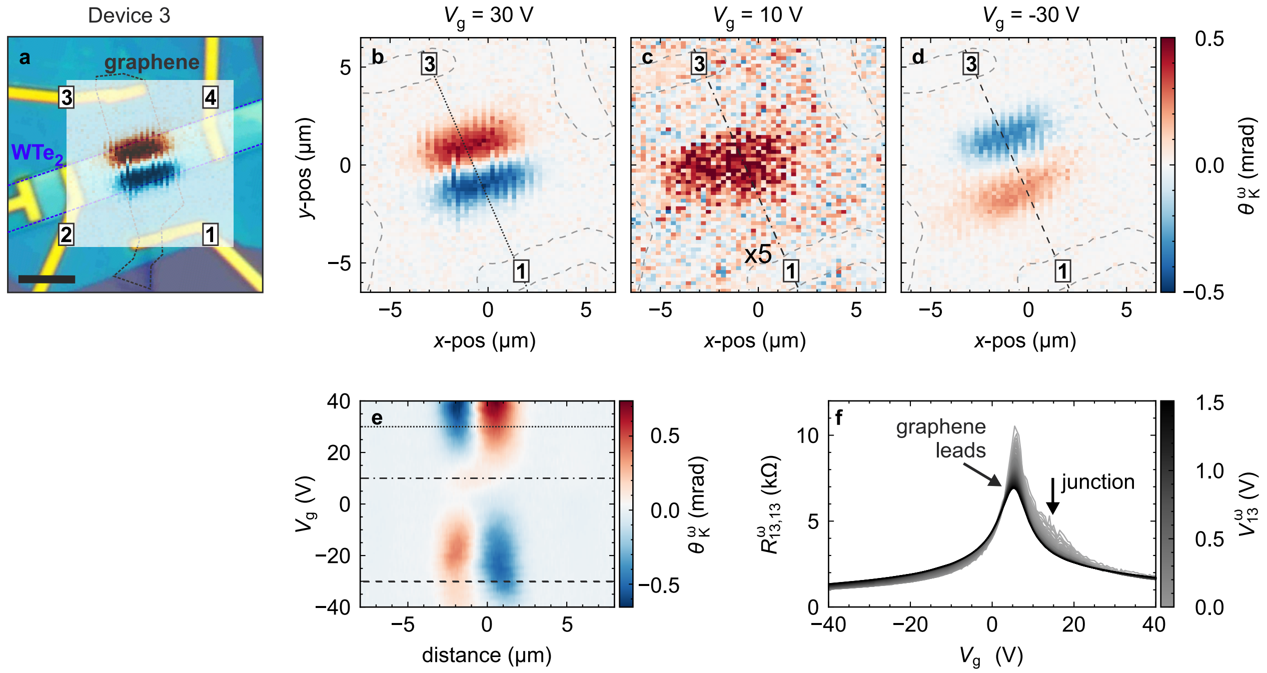

Supplementary Fig. S4. **Extended data of a third device.** a) Optical microscopy image of a heterostructure comprised of graphene (black dashed line), WTe_2 (blue dashed line), and hBN capping. Scale bar is 5 µm. The overlay (shaded area) shows the current-induced KR signal at the junction using a colour code as in panel (d), for a current applied between the contacts labelled 1 and 3. b)-d) Spatially-resolved magneto-optic KR signal under ac current flow along the graphene stripe for (b) $V_\mathrm{g} = 30\,\mathrm{V}$, (c) $V_\mathrm{g} = 5\,\mathrm{V}$, (d) $V_\mathrm{g} = -30\,\mathrm{V}$. The Kerr angles are measured at the fundamental frequency ω of the alternating bias current. Grey dashed lines highlight the metal electrodes. The data in c) is scaled by a factor of 5 for clarity. $T_\mathrm{bath} = 4.2\,\mathrm{K}$, $V_{13}^{\omega} = 3\,\mathrm{V}$. e) Spatially-resolved KR θ_K^ω across the graphene/WTe_2 heterojunction as a function of applied gate voltage. Dotted, dash-dotted and dashed lines correspond to profiles indicated in panels b), c), and d), respectively. f) Two-terminal resistance $R^\omega_{13,13}$ of the graphene stripe measured between contacts 1 and 3 of the device in panel (a), at $T_\mathrm{bath} = 4.2\,\mathrm{K}$ using an ac voltage that increases stepwise up to 1.5 V.

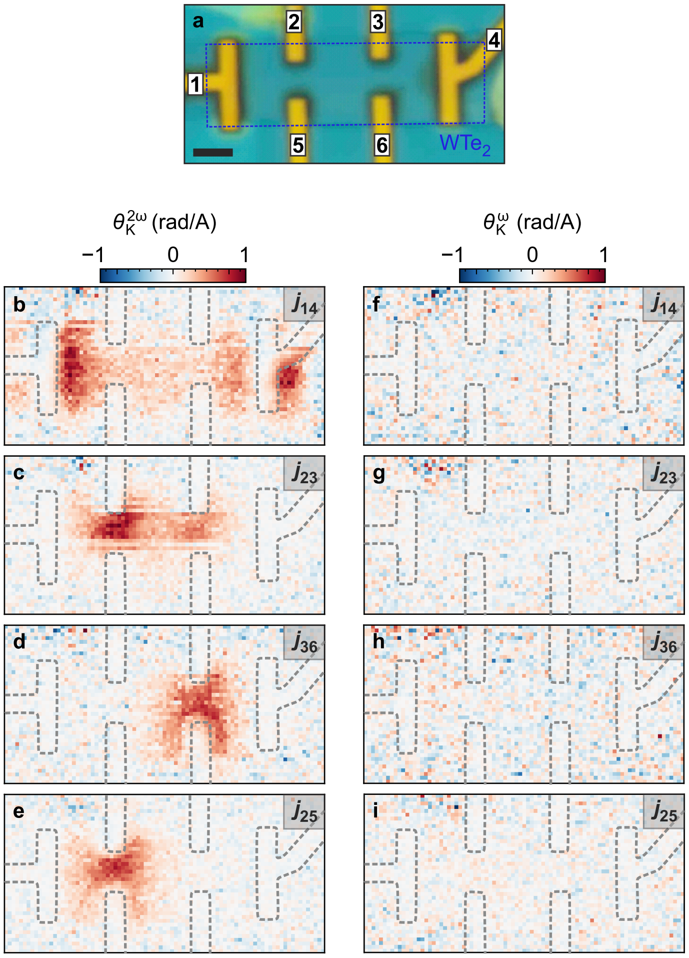

Supplementary Fig. S5. **Mapping of local charge current and absence of current-induced Kerr rotation along different crystal axes in bare WTe_2.** a) Optical microscope image of a WTe_2 Hall bar device. The long (short) axis corresponds to the a-axis (b-axis). An in-plane AC current $j(\omega)$ was applied in different contact configuration corresponding to currents along different crystal axes. The scale bar is 2 µm. b)-e) Polarization rotation detected at the second harmonic 2ω of the AC frequency (3.33 kHz). The polarization rotation reveals the local heating due the local current flow between the respective contacts used for current injection. f)-i) Current-induced Kerr rotation detected at the first harmonic ω of the AC frequency. No Kerr rotation was detectable for any of the contact configurations or crystal axes.

KR microscopy - power dependence

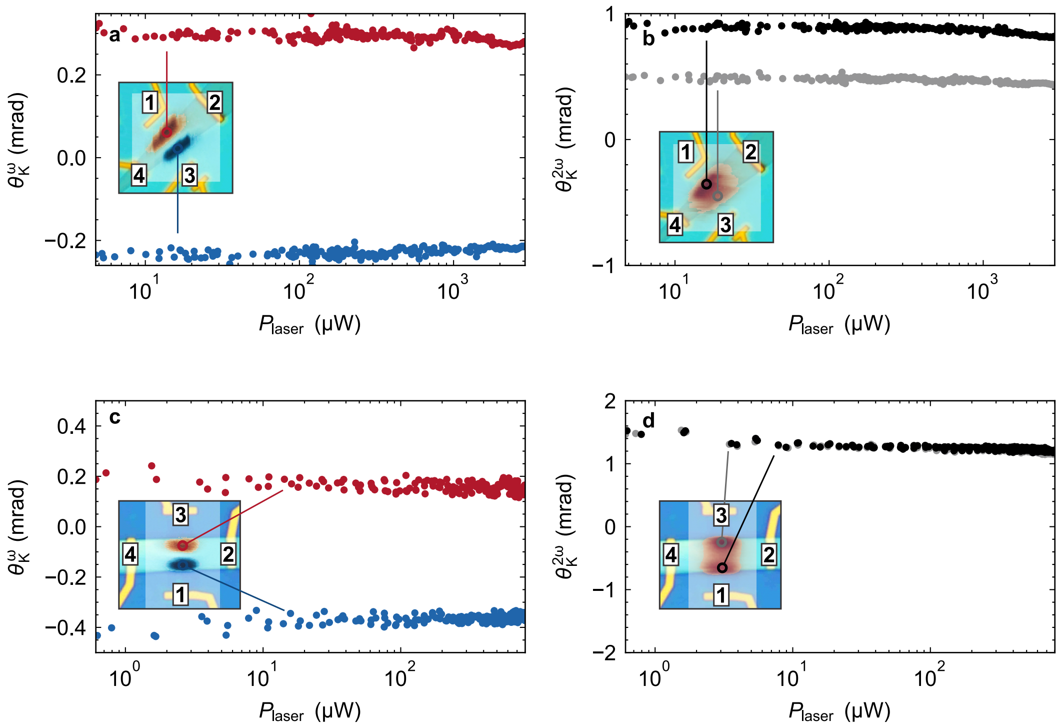

Supplementary Fig. S6. **Optical power-dependence of current-induced KR.** a) First harmonic KR signal θ_K^ω as a function of laser power P_{laser} measured at the two junction edges for a current applied along the graphene stripe (contacts labelled 1 and 3). The current-induced KR angle is independent of laser power over almost three orders of magnitude ruling out higher order effects and local photocurrents to be the origin of the local Kerr rotation. b) Corresponding second harmonic KR signal $\theta_K^{2\omega}$. c)-d) First and second harmonic KR signal as a function of laser power of a second device. The measurements were recorded at $T_{\mathrm{bath}} = 4.2\,\mathrm{K}$, $V_{13}^\omega = 2\,\mathrm{V}$, and $V_{\mathrm{g}} = -30\,\mathrm{V}$.

Theory - Derivation of the Kerr response

In this section, we present details on the Kerr response and topological extension of Fick theory. We explain the solution for the problem of the WTe_2 slab on the graphene stripe. We use natural units, $\hbar = 1$ in this supplementary information.

Kerr response

The Kerr rotation angle in birefringent materials is (for an analogous formula in bulk materials, see F.J. Kahn *et al.*, Phys. Rev. **186**, 891 (1969))

$$\tan(\theta_K) = -\text{Re}\frac{\sigma_H^{2D} + \sigma_{\text{BF}}^{2D}\sin(2\alpha)}{\sigma^{2D}}. \tag{1}$$

This equation directly follows from classical electrodynamics in the limit when the gyrotropic material is thin as compared to the wavelength of light. Without loss of generality the conductivity tensor in the x-y plane can be written as

$$\underline{\sigma} = \begin{pmatrix} \sigma + \sigma_{\text{BF}} & \sigma_H \\ -\sigma_H & \sigma - \sigma_{\text{BF}}. \end{pmatrix}, \tag{2}$$

where α is the angle of polarization of the vertically incident light. In this limit, $\sigma^{2D}(x,y) = \int_0^h dz\sigma(x,y,z)$ is the sheet conductance. The Hall and anisotropy terms σ_H^{2D} and $\sigma_{\text{BF}}^{2\text{D}}$ are defined analogously. Note that all conductivities entering the Kerr response ought to be evaluated at the optical frequency ν. We highlight that in anisotropic materials a deviation from the perfect $\sin(2\theta)$ angular dependence on the angle of polarization in Eq. (1) may enter through microscopic effects entering optical conductivities, for an example in the context of Weyl candidate materials see, e.g.,[53]. This may be the source of the non-trivial angular behavior observed in supplementary Fig. S8. However, to the best of our knowledge, all birefringent effects vanish from $\int_0^{2\pi} d\alpha\theta_K \sim \sigma_H$, which is exclusively a measure of (current-induced) time reversal symmetry breaking. Since our experimental data has prominent non-zero average of the Kerr angle, in the remainder of this supplementary information, and in the main text, we disregard the contribution of $\sigma_{\text{BF}}^{2\text{D}}$ and concentrate on the effect of time reversal symmetry breaking.

We next establish the relationships between θ_K, magnetization and the Berry curvature dipole, as presented in Eq. (1) of the main text. First, following the classic literature on the anomalous Hall effect[37]

$$\sigma_H(\nu) = \frac{-R_1\langle S_z\rangle}{\sigma_{xx}\sigma_{yy}}, \tag{3}$$

where R_1 is a phenomenological constant (more generally: a function of ν) and $\sigma_H \ll \sigma_{xx,yy}$ are 3D optical conductivities. Together with Eq. (1) this establishes the left proportionality in Eq. (1) of the main text, i.e. $\langle S_z\rangle \propto \theta_K$. Microscopically, the average magnetization is $\langle S_z\rangle = \int d^3p m_z(\mathbf{p})f(\mathbf{p})/(2\pi)^3$, where $\mathbf{m}(\mathbf{p})$ is the expectation value of the spin operator with respect to Bloch states with momentum momentum $\mathbf{p}$ and dispersion $\epsilon(\mathbf{p})$. In general, the orbital magnetization may also contribute to $\langle S_z\rangle$. It vanishes in equilibrium due to time reversal symmetry, when the distribution function $f(\mathbf{p})$ is the Fermi-Dirac function $f_{\text{FD}}(\epsilon(\mathbf{p}))$. However, in the presence of a driving field $f(\mathbf{p}) = f_{\text{FD}}(\epsilon(\mathbf{p} - \tau e\mathbf{E}(\omega)))$ (τ is the current relaxation time and short as compared to $1/\omega$), so that

$$\sigma_H(\nu,\omega) = \frac{R_1\tau e M_{jz}}{\sigma_{xx}\sigma_{yy}}E_j(\omega), \quad \text{where} \quad M_{jk} = \int \frac{d^3p}{(2\pi)^3}m_k(\mathbf{p})\frac{\partial f_{\text{FD}}(\epsilon(\mathbf{p}))}{\partial p_j}. \tag{4}$$

Now we discuss the right relationship in Eq. (1) of the main text, i.e. $\theta_K \propto D_{jz}^{(\Omega)}j_j(\mathbf{x})$. In contrast to the phenomenological Eq. (3), this relationship is microscopic and given by the evaluation of the Hall response $\sigma_H(\nu,\omega) = \int d^3p\Omega_z f(\mathbf{p})/(2\pi)^3$ [39]

$$\sigma_H(\nu,\omega) = e^3\tau D_{jz}^{(\Omega)}E_j(\omega), \quad \text{where} \quad D_{jk}^{(\Omega)} = \int \frac{d^3p}{(2\pi)^3}\Omega_k\frac{\partial f_{\text{FD}}(\epsilon(\mathbf{p}))}{\partial p_j}. \tag{5}$$

Here $\Omega_k(\mathbf{p})$ is the Berry curvature and $D^{(\Omega)}_{jk}$ the Berry curvature dipole. This relationship is closely related to the calculation of the non-linear Hall response [32] and valid in the following two cases: (i) when ν is smaller than the bandwidth and larger than the relaxation rate; (ii) when ν is larger than the bandwidth only in the case of effective two-band models.

While this concludes the derivation of the proportionalities $\langle S_z\rangle \propto \theta_K \propto D^{(\Omega)}_{jz}E_j(\omega)$ (the left proportionality having been discussed above), we are left with the question whether $\langle S_z\rangle \propto D^{(\Omega)}_{jz}E_j(\omega)$ holds. Comparison of Eqs. (4) and Eq. (5) elucidates that this is only true if $M_{jk} \propto D^{(\Omega)}_{jk}$. While magnetization $m_k(\mathbf{p})$ and Berry curvature $\Omega_k(\mathbf{p})$ have the same symmetry properties, and are proportional for simple, isotropic Hamiltonians, e.g. the linearized Weyl Hamiltonian, they are generally not proportional. Thus $M_{jk} \not\propto D^{(\Omega)}_{jk}$ and $\langle S_z\rangle \not\propto D^{(\Omega)}_{jz}E_j(\omega)$. The reason for the mismatch is the left proportionality in Eq.(1), which is only phenomenological, Eq. (3), and not precise - the current-induced MOKE measurement is a probe of spin accumulation only in simplified models but in more generality a microscopically justified measure of the Berry-curvature under the more generic assumption outlined above.

Despite the fact that generally, $M_{jk} \not\propto D^{(\Omega)}_{jk}$ (in the sense of a proportionality between tensors), a proportionality $M_{jk}\propto D^{(\Omega)}_{jk}$ for each given tensor component is well defined in the following sense: As both tensors M_{jk} and $D^{(\Omega)}_{jk}$ have the same symmetry properties, given tensor components (in our case M_{zz} and $D^{(\Omega)}_{zz}$ are most relevant) display the same asymptotic behavior for weak symmetry breaking perturbation (in our case related to broken $b \to -b$ symmetry).

Topological Fick theory

In this section we develop an effective semiclassical theory which allows to calculate the local Hall response $\sigma_H(\nu,\omega;\mathbf{r}) = -\int d^3p\Omega_z f(\mathbf{p},\mathbf{r})/(2\pi)^3$. To this end, we derive an effective topological diffusion theory, which contains a set of 7 differential equations for density, current density and Berry curvature density. These are defined as

$$n = \int (dp) f(\mathbf{p},\mathbf{r}), \tag{6}$$

$$\mathbf{j} = \int (dp)\mathbf{v} f(\mathbf{p},\mathbf{r}), \tag{7}$$

$$\boldsymbol{\varpi} = \int (dp)\boldsymbol{\Omega} f(\mathbf{p},\mathbf{r}), \tag{8}$$

respectively, where $(dp) = d^dp/(2\pi)^d$ is the measure of momentum integrals, $\mathbf{v}$ is the derivative of the dispersion relation $\epsilon(\mathbf{p})$ and $\boldsymbol{\Omega} = \boldsymbol{\Omega}(\mathbf{p})$ is the Berry curvature.

The Boltzmann equation describing a generic topological material is given by

$$\dot{f} + \dot{\mathbf{r}}\partial_{\mathbf{r}} f + \dot{\mathbf{p}}\partial_{\mathbf{p}} f = St[f], \tag{9}$$

where $St[f]$ denotes the collision integral which we treat in the relaxation time approximation, $\dot{\mathbf{p}}$ is given by the external force (Newton's law) and $\dot{\mathbf{r}} = \mathbf{v} + \boldsymbol{\Omega}\times\dot{\mathbf{p}}$.

To derive the topological Fick diffusion theory, we assume fast equilibration of the distribution function $f(\mathbf{p},\mathbf{r},t)$, and replace, $\int(dp)v_iv_j\partial_{x_j}f$ with $\partial_{x_j}nD^{ij}/\tau$, where $D^{ij} = \langle v^iv^j\rangle_{\mathrm{FS}}\tau$ and $\langle\dots\rangle_{\mathrm{FS}}$ is the average over the Fermi surface. Similar replacements occur in other quantities. Then, Fick's first law (i.e. the diffusive relationship between current and density) follows from multiplying the Boltzmann equation with v_i and subsequent integration $\int(dp)$ of the entire equation (assuming that the current dynamics is slow with respect to τ). The second law is the continuity equation obtained by just integrating the Boltzmann equation. The topological extension is obtained by multiplying the equation by $\boldsymbol{\Omega}$ before integration. This concludes the derivation of Fick diffusion theory presented in the main text.

Next, we comment on the source term for the coupled heterostructure. Using $w_{t,\mathbf{r},\mathbf{p}}$, and $g_{t,\mathbf{r},\mathbf{p}}$ to denote the time t, position $\mathbf{r}$ and momentum $\mathbf{p}$ dependent distribution function in WTe_2 and graphene, respectively, we consider the following corrections to the collision integral

$$\delta St_w[w,g] = -\int \frac{d^2p'}{(2\pi)^2}\omega_{\mathbf{p},\mathbf{p}'}[w_{t,\mathbf{r},\mathbf{p}} - g_{t,\mathbf{r},\mathbf{p}'}]c\delta(z)\chi(x,y), \tag{10a}$$

$$\delta St_g[w,g] = -\int \frac{d^3p'}{(2\pi)^3}\omega_{\mathbf{p}',\mathbf{p}}c[g_{t,\mathbf{r},\mathbf{p}} - w_{t,\mathbf{r},\mathbf{p}'}]_{z=0}\chi(x,y). \tag{10b}$$

It describes the particle exchange at the interface (in an idealized situation, $\chi(x, y) = 1$ where graphene and WTe_2 overlap and zero otherwise). This explains the appearance of the delta function $\delta(z)$ (the constant c is the size of the unit cell of WTe_2 in z-direction). For simplicity, we will model $\omega_{\mathbf{p},\mathbf{p}'} = \omega_0 \delta_{\mathbf{p}_\parallel,\mathbf{p}'} + \omega_1$ (accounting for completely momentum conserving and completely momentum scrambling contributions, respectively). Note that all perpendicular plane waves in WTe_2 with arbitrary p_z couple to the graphene, as they all have some weight on the $z = 0$ surface.

With these assumptions, the collision integral leads to the following modifications of the continuity equation and the Fick's laws:

(i) The continuity equation (essentially Fick's second law) obtains an addition which is given by particle density exchange between graphene and WTe_2. This term stems from both ω_0, ω_1 and is responsible for the charge transfer in equilibrium. As we are interested in the non-equilibrium transport setting, we perturb about this induced equilibrium state. We effectively account for it by imposing different equilibrium densities underneath the junction and in the graphene leads, and therefore will not discuss the effect of ω_1, explicitly.

(ii) The First Fick's law, obtained by first multiplying the Boltzmann equation with v_i and then integration, contains additional terms of current exchange at the interface,

$$\frac{D_w^{ij}}{\tau_w}\partial_j n_w = -\frac{j_w^i}{\tau_w} + \alpha_w \frac{\bar{D}_w^{ij}\partial_j n_g}{\tau_w}\delta(z), \tag{11}$$

$$\frac{D_g^{ij}}{\tau_g}\partial_j n_g = -\frac{j_g^i}{\tau_g} + \alpha_g \frac{\bar{D}_g^{ij}\partial_j n_w}{\tau_g}|_{z=0}. \tag{12}$$

Here, $\alpha_{w,g}$ are constants proportional to ω_0. The constants $\bar{D}_w^{ij} = \langle v_w^i v_g^j \rangle_{(\text{FS of graphene})}\tau_w$, $\bar{D}_g^{ij} = \langle v_g^i v_w^j \rangle_{(\text{FS of WTe}_2)}\tau_w$ are generated from Eqs. (10) using the assumption of fast equilibration. Note that $\bar{D}^{ij}$ is opposite for electron or hole carriers in graphene. This is the origin of the ambipolar nature of the effect discussed in the main text.

In the following, we simplify these equations, by setting $D_w^{ij} = D\delta^{ij}$, $D_g^{ij} = D_g\delta^{ij}$, $\alpha_w \bar{D}_w^{ij} = \bar{D}_w \delta^{ij}$, $\alpha_g \bar{D}_g^{ij} = ca\bar{D}_g\delta^{ij}$ (where c is a UV length scale and D_g the diffusion constant in graphene). We also fix $\bar{D}_w > 0$ and absorb the sign of $\bar{D}_w$ into the density: Ultimately, the source term for the current becomes $\pm\bar{D}_w\partial_{\mathbf{x}} n_g$ (with the sign being positive/negative in conduction/valence band) - this is the origin of the coupling to the imbalance current discussed in the main text (there, the subscript "$_w$" is omitted from $\bar{D}_w$).

Finally, we briefly consider the correction to the topological extension

$$D_{ji}^{(\Omega)}\partial_j n_w = -\frac{\varpi_i}{\tau_w} + \bar{\alpha}\frac{\bar{D}_{ji}^{(\Omega)}\partial_j n_g}{\tau_w}\delta(z). \tag{13}$$

The symmetry properties of both $D_{ji}^{(\Omega)}$ and $\bar{D}_{ji}^{(\Omega)}$ are the same as those of λ_{ji} discussed in the main text - the only non-zero contribution is $i = j = z$, but there is no current in graphene in z-direction. Hence, the correction in Eq. (13) vanishes.

Imbalance decay

We briefly remind the reader about transport in pure graphene. Near charge neutrality, there are actually two types of carriers: electrons, which are excitations in the conduction band, and holes, i.e. excitations in the hole band. We denote the associated charge densities n_e and n_h respectively. While overall charge conservation prohibits a decay of the total charge $q = n_e - n_h$, the charge imbalance $n_I = n_e + n_h > 0$ may relax and we now introduce a theory of this decay. We stress that the electron and hole density are space-dependent even in equilibrium (due to local charge transfer under the WTe_2 stripe).

Imbalance relaxation occurs through decays $e^- \to e^- + e^- + h^+$, and is therefore a non-linear function of densities. At finite doping, the minority carrier density vanishes in equilibrium and zero temperature: In this case $n_I = |q|$, while in general (e.g. not at equilibrium) $n_I \geq |q|$. All of these aspects are encoded in the following addition to the continuity equation of the imbalance density n_I

$$\dot{n}_I - D_g\nabla^2 n_I = -\frac{n_I^2 - q^2}{2q_0^2}\frac{n_I}{\tau_{\text{rec}}}. \tag{14}$$

Here, τ_{rec} is the recombination rate, which is typically macroscopically large. The charge q_0 is the externally imposed charge density, which is modelled by a piecewise constant function.

Then, we linearize these equations around the equilibrium value.

We now return to the heterostructure. The coupled equations of interest between graphene and WTe_2 are thus

$$-D\nabla^2 n_w = -\alpha\partial_x[\bar{D}_w\partial_x n_I]\delta(z), \tag{15}$$

$$-D_g\partial_x^2 n_I = -\frac{n_I - |q|}{\tau_{\rm rec}} - \partial_x[a\bar{D}_g\partial_x n_w]_{z=0}, \tag{16}$$

supplemented with charge conservation, which implies $\dot{n}_w + \dot{q} + \nabla(j_w + j_q) = 0$. We use the notation for currents and densities $j_w = -D\nabla n_w, j_I = -D_g\nabla n_I$, and $j_q = -D_g\nabla q$, where n_w is the density in WTe_2. For simplicity, we consider time- and y-independent solutions which is the reason for dropping time and ∂_y gradients. We further apply a transport current, such that charge conservation implies $j_q + \int_0^h dz j_{W,x} = j_0 = \text{const.}$.

We will assume that the hybridization $\bar{D}_w = \bar{D}_g = 0$ at the boundary $x = \pm l/2$, and that it sets on in a smooth rapid fashion beyond the boundary (i.e. it is spatially dependent). At the same time, diffusion constants are assumed spatially independent.

In these equations we have focussed on the effect of momentum transfer between graphene and WTe_2, i.e. a current in WTe_2 imposes an imbalance current in graphene and vice versa. We exploit that a non-zero momentum transfer can always be expected at the interface and it is the minimal ingredient for the Kerr response. Charge transfer, which might also be present but does not affect current injection and the Kerr response, is neglected here.

Green's function in WTe_2

Let $G(x, x'; z, z')$ be the Green's function, i.e. $\nabla^2 G(x, x'; z, z') = \delta(x - x')\delta(z - z')$ of the Laplacian inside the WTe_2 sample, $(x, z) \in (-l/2, l/2) \times (0, h)$ with von-Neumann boudnary condition $\mathbf{j} \cdot \hat{\mathbf{n}} = 0$. (Note that this does not impede tunnelling into or out of the sample.)

The Green's function of the 2D Laplacian is $G_0(x, z) = \ln(x^2+z^2)/(4\pi)$. The no outflux boundary conditions can be imposed by an infinite sequence of (mirror) charges located at $(x_n, z_m) = (2nl+x', 2mh+z')$, $(x_n, z_m) = ((2n+1)l-x', 2mh+z')$, $(x_n, z_m) = ((2n+1)l-x', 2mh+z')$, $(x_n, z_m) = (2nlx', 2mh-z')$ and $(x_n, z_m) = ((2n+1)l-x', 2mh-z')$.

Deep inside the stripe of height $h \ll l$, the Green's function is dominated by charges at $x = x'$ in which case

$$G(x, x'; z, z') = \frac{1}{4\pi}\left\{\ln\left[\cosh(\frac{\pi(x-x')}{h}) - \cos(\frac{\pi(z-z')}{h})\right] + \ln[\cosh(\frac{\pi(x-x')}{h}) - \cos(\frac{\pi(z+z')}{h})]\right\}. \tag{17}$$

Assuming both x and x' at distances larger than h from the boundary, we obtain

$$\int_0^h dz\partial_x G(x, x'; z, 0) = \frac{\text{sign}(x-x')}{2}, \tag{18a}$$

$$\int_0^h dz\partial_z G(x, x'; z, 0) = \frac{1}{2\pi}\ln\left(\frac{\cosh(\pi(x-x')/h)+1}{\cosh(\pi(x-x')/h)-1}\right). \tag{18b}$$

For trial functions, which are smooth on the scale h one may further approximate

$$\partial_x G(x, x'; z, 0) \simeq \frac{\text{sign}(x-x')}{2h}, \tag{18c}$$

$$\int_0^h dz\partial_z G(x, x'; z, 0) \simeq \frac{h}{\pi}\delta(x-x'). \tag{18d}$$

Solution of diffusion equation in WTe_2

Using the Green's function, the solution of the diffusion equation, Eq. (15), is

$$n_w(x, z) = \int_{-l/2}^{l/2} dx' G(x, x'; z, 0)\partial_{x'}[\bar{D}_w\partial_{x'}n_I]/D, \tag{19a}$$

which implies in terms of currents

$$\mathbf{j}_w = \int_{-l/2}^{l/2} dx'\nabla G(x, x'; z, 0)\partial_{x'}\left[\frac{\bar{D}_w}{D_g}j_I(x')\right]. \tag{19b}$$

We use this, as we determine the total currents in the sample,

$$
\begin{aligned}
j_x^{\rm tot} \equiv \int_0^h dz j_{w,x} &= \int_{-l/2}^{l/2} dx' \int_0^h dz \partial_x G(x,x';z,0)\partial_{x'}\left[\frac{\bar{D}_w}{D_g} j_I(x')\right] \\
&= \int_{-l/2}^{x} \frac{dx'}{2}\partial_{x'}\left[\frac{\bar{D}_w}{D_g} j_I(x')\right] - \int_{x}^{l/2} \frac{dx'}{2}\partial_{x'}\left[\frac{\bar{D}_w}{D_g} j_I(x')\right] \\
&= \frac{\bar{D}_w}{D_g} j_I(x),
\end{aligned} \tag{19c}
$$

$$
\begin{aligned}
j_z^{\rm tot} \equiv \int_0^h dz j_{w,x} &= \int_{-l/2}^{l/2} dx' \int_0^h dz \partial_z G(x,x';z,0)\partial_{x'}\left[\frac{\bar{D}_w}{D_g} j_I(x')\right] \\
&= \int_{-l/2}^{l/2} dx' [G(x,x';h,0) - G(x,x';0,0)]\partial_{x'}\left[\frac{\bar{D}_w}{D_g} j_I(x')\right] \\
&\simeq \frac{h}{D_g \pi}\partial_x[\bar{D}_w j_I].
\end{aligned} \tag{19d}
$$

Here, we made use of Eqs. (18), which are valid at positions x, x' which are distant h or larger from the boundary. We thus found that the Kerr angle is directly proportional to $\partial_x j_I(x)$. A smooth $\bar{D}_w j_I(x)$ allows to write

$$
j_x(x,z) \simeq \frac{\bar{D}_w}{h D_g} j_I(x). \tag{19e}
$$

Note that $\bar{D}_w j_I$ varies on the scale of the imbalance relaxation length, which is macroscopic and substantially exceeds h. Therefore, $|j_z| \ll |j_x|$.

It is furthermore obvious, that only those current configurations with $\int dx \partial_x \frac{\bar{D}_w}{h D_g} j_I(x) = 0$ can satisfy the requirement of no current outflux. Since the same amount of current flows into WTe_2 and out of WTe_2 near either edge, this naturally explains the dominant antisymmetric spatial dependence of $\theta_K(x)$. At the same time, the contribution to $\theta_K(x)$ with a non-zero spatial average must stem from a different, weaker effect.

Solution of the diffusion equation in graphene

We use Eq. (19) in the diffusion equation for graphene, which becomes

$$
-D_{\rm eff}\nabla^2 n_I = -(n_I - |q|)/\tau_{\rm rec}, \tag{20}
$$

where $D_{\rm eff} = D_g + \frac{a \bar{D}_g \bar{D}_w}{h D_g D}$. The solution to this equation leads to

$$
j_I = {\rm sign}(q) j_{q,0} + \bar{j}_I \frac{\cosh(x/l_{\rm rec})}{\cosh(l/2l_{\rm rec})} + \delta j_I \frac{\sinh(x/l_{\rm rec})}{\sinh(l/2l_{\rm rec})}, \tag{21}
$$

where $l_{\rm rec} = \sqrt{D_{\rm eff}\tau_{\rm rec}}$ is the mean recombination path and we express the two boundary conditions by $\bar{j}_I, \delta j_I$, where ${\rm sign}(q_0) j_{q,0} + \bar{j}_I \pm \delta j_I/2$ are the imbalance currents at $x = \pm l/2$ (here, q_0 is the equilibrium density underneath the junction).

Boundary conditions

We now model the boundary conditions on j_I with the following two main requirements

- The boundary current is odd in the externally applied current $j_{q,0}$,
- and odd under particle hole transformation.

The last point derives from the requirement that deep in the bands, $j_I \propto \mathrm{sign}(q) j_q$ and is equivalent to inverting energy about the Dirac node, i.e. $j_I \propto \mathrm{sign}(\mu - E_{\mathrm{Dirac}}) j_q$ near equilibrium. This motivates the following Ansatz

$$\bar{j}_I = \mathcal{C} \underbrace{\frac{1}{\pi} \left\{ \arctan([\mu - E_{\mathrm{out}}]\tau) + \arctan([\mu - E_{\mathrm{in}}]\tau) \right\}}_{f(\mu)} j_{q,0}, \tag{22a}$$

$$\delta j_I = \mathcal{C} \frac{\delta\mu}{2\pi} \frac{\partial}{\partial \mu} \left\{ \arctan([\mu - E_{\mathrm{out}}]\tau) + \arctan([\mu - E_{\mathrm{in}}]\tau) \right\} j_{q,0}, \tag{22b}$$

where $E_{\mathrm{out}}, E_{\mathrm{in}}$ are the energy of the Dirac point outside and inside the junction, respectively. Note that $\bar{j}_I, \delta j_I$ are boundary conditions on the correction to the imbalance density $n_I - |q| = n + p - |n - p|$, which is nothing but the minority charge. Therefore, the constant $\mathcal{C}$ becomes nonezero only when the bias voltage exceeds the distance to the Dirac node, as observed in experiment.

Kerr response

The current-induced Hall response in WTe_2 is $\sigma_H = \tau_w D_{zz}^{(\Omega)} \int_0^h dz \partial_z n_w = -\tau_w D_{zz}^{(\Omega)} j_z^{\mathrm{tot}}/D$. The Kerr angle is thus

$$\theta_K = \frac{\sigma_H}{\sigma_w^{\mathrm{tot}} + \sigma_g} \simeq \frac{-h\tau_w D_{zz}^{(\Omega)} \partial_x [\bar{D}_w j_I]/(D_g D)}{h D \nu_w}. \tag{23}$$

We can estimate the magnitude as follows

$$\theta_K \sim \frac{\bar{D}_w D_{zz}^{\Omega} \tau_\omega}{D_g D^2 \nu_w l_{\mathrm{rec}}} j_{q,0} \sim \frac{\Omega}{v_F^2 \nu_w} \frac{1}{\ell l_{\mathrm{rec.}}} j_{q,0} \sim \frac{1}{p_F^2 \ell l_{\mathrm{rec}}} \frac{1}{W p_F} \frac{I}{e E_F}. \tag{24}$$

The constants entering this equation are ν_w (density of states in WTe_2), p_F/E_F (Fermi momentum/energy in WTe_2), W (slab width), ℓ (mean free path). Here, some constants of order unity have been dropped: the transparency of the graphene and WTe_2 junction and $\mathcal{C}$ (for the contribution due to j_I). For $I \sim 1\,\mathrm{mA}$, $E_F \sim 100\,\mathrm{meV}$ (in WTe_2), the factor is $I/(eE_F) \sim 40$. Choosing realistic numbers for the other parameters $p_F \ell \sim 10$, $W p_F \sim 100$, $l_{\mathrm{rec}} p_F \sim 100$ lead to $\theta_K \sim$ mrad at $I = 1\,\mathrm{mA}$.

Fit to experiment

It is important to keep in mind that the local $j_z^{\mathrm{tot}}(x)$ in WTe_2 can only account for the Kerr response which average to zero over the entire sample. Differently stated: The condition that current can only leave WTe_2 at the interface with graphene does not allow for a non-zero $\int dx j_z^{\mathrm{tot}}(x)$. Therefore, we here subtract the mean $\bar{\theta}_K(V_{\mathrm{g}}) = \int dx \theta_K(x, V_{\mathrm{g}})$ from the measured Kerr response at each value of gate voltage, see Fig. S7

Motivated by the theoretical relationship, Eqs. (19),(21),(23), we expand in small $l \ll l_{\mathrm{rec}}$, and fit the data to the following simplified functional form

$$\theta_K = \frac{\partial \left[g(x, l_{\mathrm{hyb}})^2 \left(|V_{\mathrm{g}}| \left(A - Bx^2 \right) f(V_{\mathrm{g}}) + Cx \right) \right])}{\partial x}, \tag{25}$$

where we set the position of the Dirac node outside (underneath) the junction $E_{\mathrm{out}} = 0$ ($E_{\mathrm{in}} = 24V$, based on the position of the second maximum in the two-point resistance). Note the additional factor of V_{g} which also derives from the relationship between current and voltage in the regime of sufficiently large bias.

The fit parameters are obtained using the Levenberg-Marquardt algorithm implemented in *Mathematica* and reported in Tab. I. The comparison to the experimental data, Fig. S7 highlights that major aspects are reproduced, particularly the ambipolar nature of the effect and the sign change as a function of position.

Variable	Estimate	Standard Error
A [mrad * l/V]	-0.0072943	0.0000798469
B [mrad / Vl]	-0.04437	0.00105311
C [mrad]	-0.0304106	0.00215033
l_{hyb} [l]	0.322342	0.00682202
τ [1/V]	0.200372	0.0110157

TABLE I. Fit parameters of Eq. (25), where $l = 5\,\mu\text{m}$ is the length of the junction. Note that τ enters as $\mu\tau$ in $f(\mu)$. At the same time, $\mu = \text{const.}V_{\text{g}}$, where empirically const. $\sim 1/200$ (i.e. 1 V of gate voltage changes the chemical potential by 5 meV). The presented value for τ does not accommodate for the empirical correction factor, but physically our best fit $1/\tau \sim 5\,\text{V}$ corresponds to a physical decay rate $1/\tau \sim 25\,\text{meV}$, which is realistic.

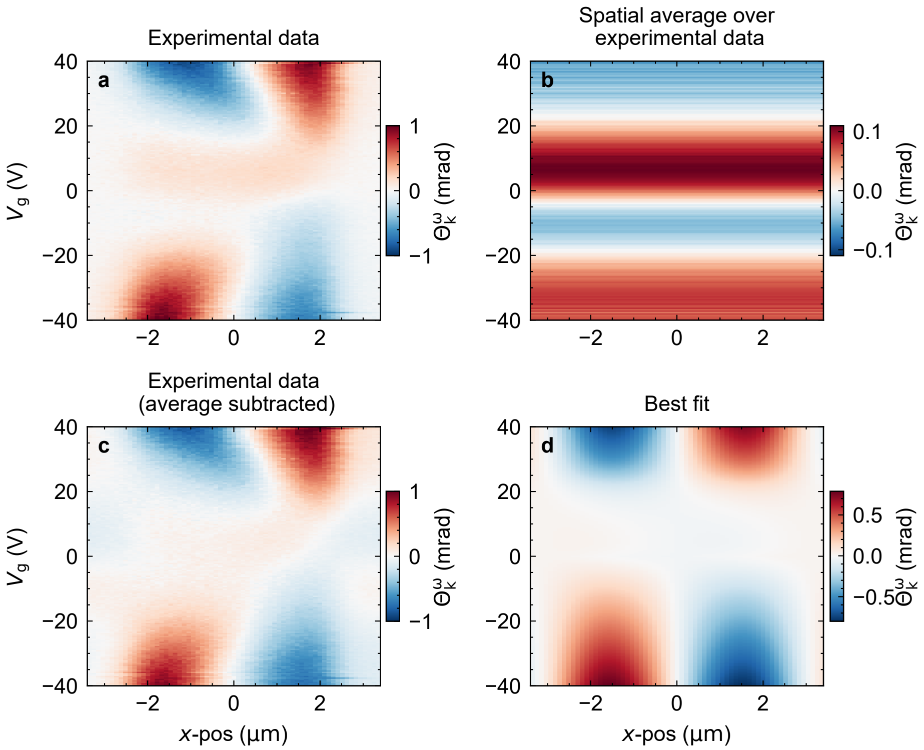

Supplementary Fig. S7. **Theory of current-induced KR in WTe_2.** (a) The experimental Kerr data, (b) the spatial average over the latter, (c) the difference between the data in panels *(a)* and *(b)*, (d) theoretical fit to *(c)* (paramters are given in Tab. I). Panels (a) and (d) are also reported in Fig. 5 of the main text.

Angular dependent Kerr measurements

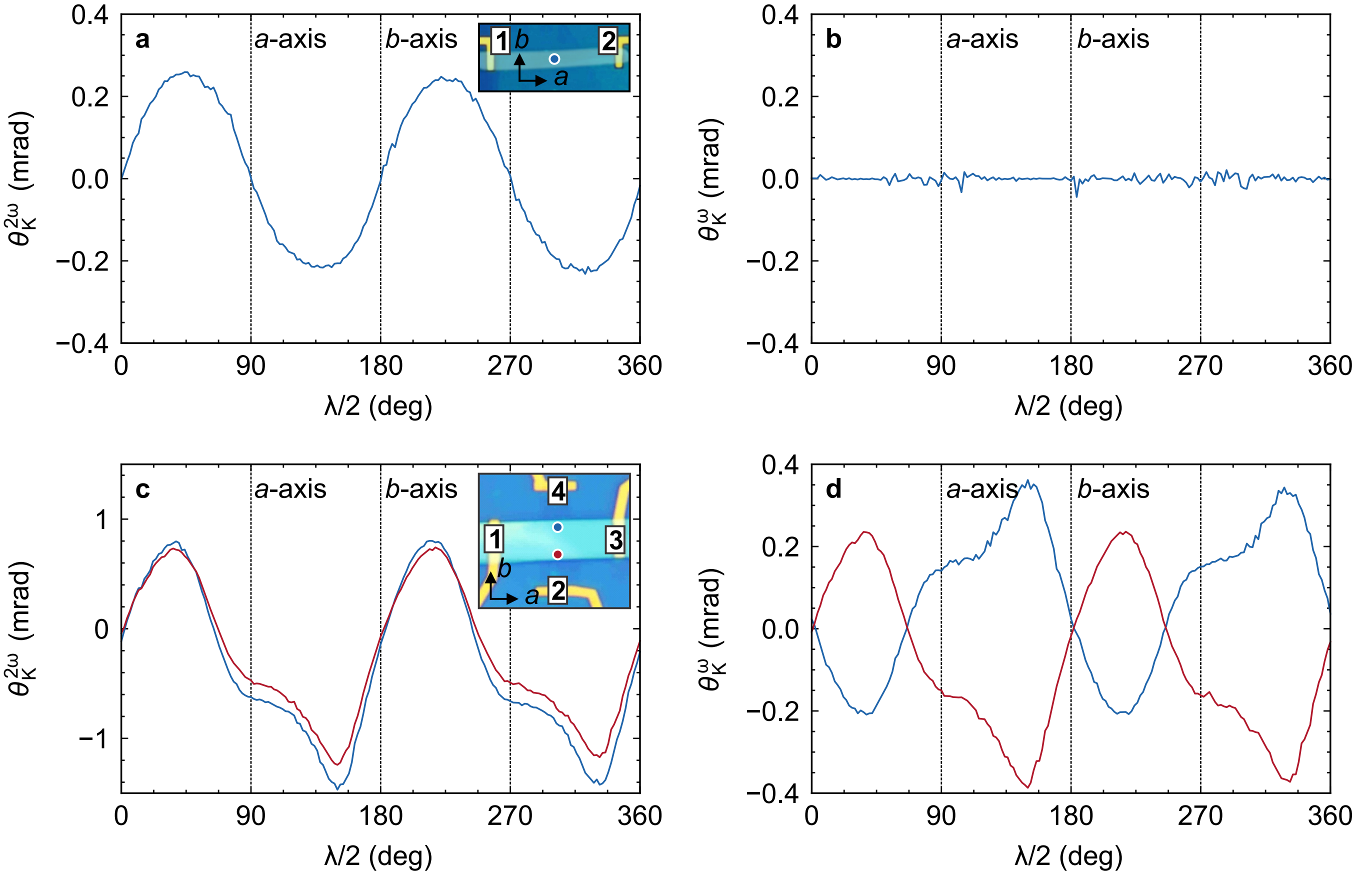

Supplementary Fig. S8. **Kerr rotation as function of the polarisation angle of the probe light.** Current-induced polarization rotation and Kerr rotation of a bare WTe_2 film measured at the second (a) and first harmonic (b), respectively. The current is passed between the contacts labelled 1 and 2 (along the a-axis of the crystal, see inset). The polarization of the probe light right is rotated in the plane of the device by a half-waveplate. The blue dot in the inset denotes the position of the laser beam. (a) The polarization rotation at the second harmonic arises from birefringence and shows the expected $sin(\alpha)$ dependence (cf. Eq. 6), which averages to zero. (b) The current-induced Kerr effect is absent for all polarization angles. (c,d) Current-induced polarization rotation and Kerr rotation of a WTe_2/graphene heterostructure measured at the second (c) and first harmonic (d), respectively. The current is passed through the graphene layer using the contacts labelled 2 and 4 (see inset). The blue and red dot in the inset denote the positions of the laser beam for the blue and red curve, respectively. (c) and (d) In the heterostructure the angular dependences of the polarization and Kerr rotation are modified. In particular, the Kerr rotation in (d) has a finite angular average, which indicates a finite current-induced magnetization.
Importantly, the fact that the graphene/WTe_2 heterostructure displays a finite angular average of both, the first and second harmonic Kerr signal, is a strong hint that the signal originates from current-induced spin polarisation.

Experimental setup

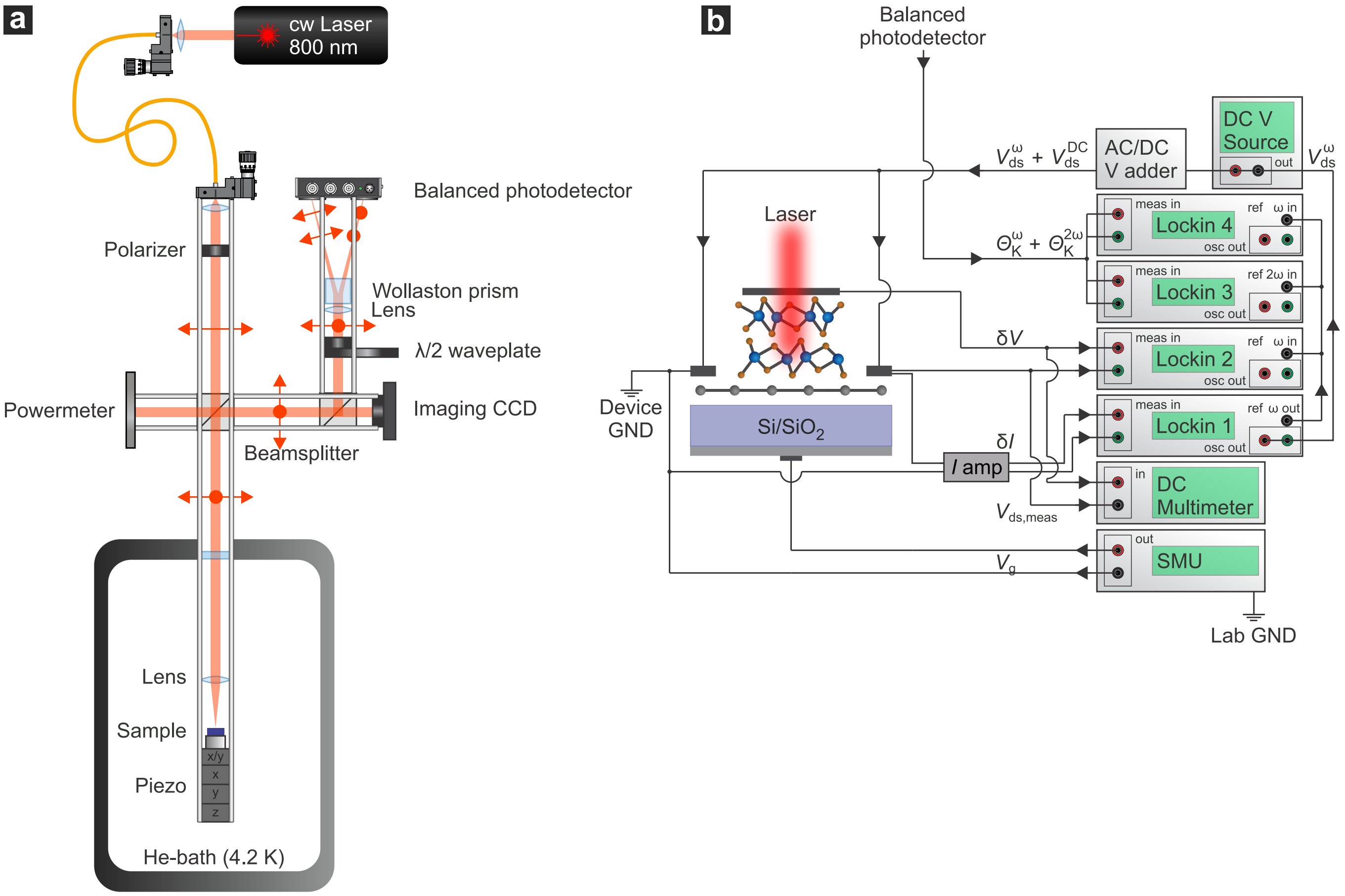

Supplementary Fig. S9. **Opto-electronic setup.** a) Schematic of the used confocal dip-stick microscope with a sample bath-temperature of 4.2 K. A linearly polarised cw-laser at $\lambda_{\text{laser}} = 800\,\text{nm}$ is focused onto the sample with a diffraction-limited spot-size of $\sim 800\,\text{nm}$. The reflected beam is guided through a 50:50 beamsplitter, a half-wave plate, a Wollaston prism, and focused onto an amplified balanced photodetector. For spatially-resolved photocurrent measurements, the laser is chopped at a frequency of 3.33 kHz. Spatially-resolved scanning is performed by moving the sample with a xy-piezo scanner mounted on top of a xyz-piezo stepper. b) Schematic electronic setup for the readout of current-induced KR, tunnelling, and photocurrent measurements. A small AC bias voltage V_{ds}^{ω} (1 mV at 77 Hz) is added to a DC bias voltage $V_{\text{ds}}^{\text{DC}}$ which are applied between source and drain contacts (e.g. across graphene or across the graphene/WTe_2 junction). The resulting change in current flow is pre-amplified and monitored with a lock-in amplifier at the fundamental frequency ω. Concurrently, the AC and DC voltage drop across the WTe_2 (or the junction) is measured using a second lockin amplifier and a DC multimeter. As a function of applied gate voltage V_{g} (source/measure unit SMU) and bias voltage $V_{\text{ds}}^{\omega} + V_{\text{ds}}^{\text{DC}}$, the KR signal is measured by decomposing the output of the balanced phtotodetector into its first two harmonic components θ_K^{ω} and $\theta_K^{2\omega}$ using two additional lock-in amplifiers.
