# Supplementary material for: Berry curvature-induced local spin polarisation in gated graphene/WTe2 heterostructures
Source: Nat Commun. 2022 Jun 7;13:3152. doi: 10.1038/s41467-022-30744-3 (PMC9174237; doi:10.1038/s41467-022-30744-3)
Supplement: Supplementary file 3 — Source Data [file 41467_2022_30744_MOESM3_ESM.zip › WTe2_Graphene/README.docx]

Berry curvature-induced local spin polarization in gated graphene/WTE2 heterostructures – Data availability and Metadata

This Document lists all Data use in the main or supplementary. All measurements have given a unique Identifier UID. A list of all relevant fabricated sample can be found in the end of this document as well. All data is stored in .h5 format

**Figure 1:**

| Explanation | AC transport | Spatial Map of Gated Kerr Signal on first harmonic | Linescan of Gated Kerr Signal | Optical Picture |
| --- | --- | --- | --- | --- |
| Sample | K3573 | K3573 | K3573 | K3573 |
| figure | Fig. 1 | Fig. 1 | Additional Data | Fig. 1 |
| UID | 200831_094305 | 200923_200842 | 20200916_24 | - |
|  |  |  |  |  |

**Figure 2:**

| Explanation | Kerr Signal when biasing across the junction (right) | Kerr Signal when biasing across the junction (left) | Kerr Signal when biasing graphene | Kerr Signal when biasing WTe2 |
| --- | --- | --- | --- | --- |
| Sample | K3573 | K3573 | K3573 | K3573 |
| figure | Fig. 2 | Fig. 2 | Fig. 2 | Fig. 2 |
| UID | 201005_064047 | 201006_010849 | 200923_235923 | 200905_095718 |
|  |  |  |  |  |

**Figure 3:**

| Explanation | Spatial dependent Kerr Signal for negative Voltage (-30V) | Spatial dependent Kerr Signal for negative Voltage (-30V) | Spatial dependent Kerr Signal for 7V |
| --- | --- | --- | --- |
| Sample | K3573 | K3573 | K3573 |
| figure | Fig. 3 | Fig. 3 | Additional Data |
| UID | 200923_200842 | 200923_235923 | 201002_115736 |
|  |  |  |  |

**Figure 4:**

| Explanation | Transport Data | Photocurrent | Differential Kerr map |
| --- | --- | --- | --- |
| Sample | K3581 | K3581 | K3581 |
| figure | Fig. 3 | Fig. 3 | Fig. 3 |
| UID | 20201128_01 | 20201210_08 | 20201208_01 |
|  |  |  |  |

**Figure 5:**

| Explanation | Linescan of Gated Kerr Signal | Theoretical Calculation Kerr Signal |
| --- | --- | --- |
| Sample | K3573 | Theory |
| figure | Fig. 5 | Fig. 5 |
| UID | 20200916_24 | KerrMap.txt |
|  |  |  |

Supplementary Figures:

**Suppl. Fig.1/2:**

| Explanation | Polarization dependent Raman | Bias and Gate Dependent KR microscopy |
| --- | --- | --- |
| Sample | K3573 | K3581 |
| figure | Suppl. Fig. 1 | Suppl. Fig. 2 |
| UID | K3573_Raman_polarization.txt | 20201206_03 |
|  |  |  |

**Suppl. Fig.3:**

| Explanation | Transport Data | Linescan of Kerr Angle | Gate Dependent Spatial Kerr maps with gate voltages -40V.. 40V in 5V steps. (biased over Gr.) |
| --- | --- | --- | --- |
| Sample | K3581 | K3581 | K3581 |
| figure | Suppl. Fig. 3 | Suppl. Fig. 3 | Suppl. Fig. 3, (plotted: -30V,5V,30V) |
| UID | 20201126_02 | 20201204_04 | 201205_spatial_gate_sweep_graphene  201205_110750, 201205_130926, 201205_151059, 201205_171234, 201205_191412, 201205_211542, 201205_231714, 201205_231714, 201206_011849, 201206_032015, 201206_052138, 201206_072307, 201206_092432, 201206_112601, 201206_152911, 201206_173045, 201206_193220, 201206_132737 |
|  |  |  |  |

**Suppl. Fig. 4:**

| Explanation | Transport Data | Linescan of Kerr Angle | Gate Dependent Spatial Kerr maps with gate voltages 30V -30V and 10V. (biased over Gr.) |
| --- | --- | --- | --- |
| Sample | K3105 | K3105 | K3105 |
| figure | Suppl. Fig. 4 | Suppl. Fig. 4 | Suppl. Fig. 4 (30V, -30V, 10V) |
| UID | 20201218_01 | 20210107_01 | 210107_013146, 210107_070612, 210107_032316 |
|  |  |  |  |

**Suppl. Fig. 5:**

| Explanation | Linescan of Power Dependence of Kerr rotation, both lobes can be extracted | Power Dependence of Kerr Signal on left and right lobe of the interface |
| --- | --- | --- |
| Sample | K3573 | K3581 |
| figure | Suppl. Fig. 5 | Suppl. Fig. 5 |
| UID | 20200919_02 | 201211_173227, 201211_174213 |
|  |  |  |

**Suppl. Fig. 6:**

WTe2- Hallbar, measurement of Kerr rotation for different Bias configurations. The bias configuration is indicated in “Explanation”

| Explanation | a_axis_long | a_axis_short | b_axis_right | b_axis_left |
| --- | --- | --- | --- | --- |
| Sample | Hallbar K3141 | Hallbar K3141 | Hallbar K3141 | Hallbar K3141 |
| figure | Suppl. Fig. 6 | Suppl. Fig. 6 | Suppl. Fig. 6 | Suppl. Fig. 6 |
| UID | 210202_094136 | 210202_160509 | 210202_112725 | 210202_203621 |
|  |  |  |  |  |

**Suppl. Fig. 7:**

| Explanation | Gate Dependent Line Scan of Kerr Signal |
| --- | --- |
| Sample | K3573 |
| figure | Suppl. Fig. S7 |
| UID | 20200916_24 |
|  |  |

**Suppl. Fig. 8:**

| Explanation | Polarisation Dependence Gr-WTe2 crossdevice | WTe2 Hallbar |
| --- | --- | --- |
| Sample | K3573 | K3645 |
| figure | Suppl. Fig. S8 | Suppl. Fig. S8 |
| UID | 20200921_10 | 20210322_09 |
|  |  |  |
