# Supplementary material for: Berry curvature-induced local spin polarisation in gated graphene/WTe2 heterostructures
Source: Nat Commun. 2022 Jun 7;13:3152. doi: 10.1038/s41467-022-30744-3 (PMC9174237; doi:10.1038/s41467-022-30744-3)
Supplement: Supplementary file 3 — Source Data [file 41467_2022_30744_MOESM3_ESM.zip › WTe2_Graphene/README.pdf]

Berry curvature-induced local spin polarization in gated graphene/WTE2 heterostructures – Data availability and Metadata

This Document lists all Data use in the main or supplementary. All measurements have given a unique Identifier UID. A list of all relevant fabricated sample can be found in the end of this document as well. All data is stored in .h5 format

Figure 1:

Explanation	AC transport	Spatial Map of Gated Kerr Signal on first harmonic	Linescan of Gated Kerr Signal	Optical Picture
Sample	K3573	K3573	K3573	K3573
figure	Fig. 1	Fig. 1	Additional Data	Fig. 1
UID	200831_094305	200923_200842	20200916_24	-

Figure 2:

Explanation	Kerr Signal when biasing across the junction (right)	Kerr Signal when biasing across the junction (left)	Kerr Signal when biasing graphene	Kerr Signal when biasing WTe2
Sample	K3573	K3573	K3573	K3573
figure	Fig. 2	Fig. 2	Fig. 2	Fig. 2
UID	201005_064047	201006_010849	200923_235923	200905_095718

Figure 3:

Explanation	Spatial dependent Kerr Signal for negative Voltage (-30V)	Spatial dependent Kerr Signal for negative Voltage (-30V)	Spatial dependent Kerr Signal for 7V
Sample	K3573	K3573	K3573
figure	Fig. 3	Fig. 3	Additional Data
UID	200923_200842	200923_235923	201002_115736

Figure 4:

Explanation	Transport Data	Photocurrent	Differential Kerr map
Sample	K3581	K3581	K3581
figure	Fig. 3	Fig. 3	Fig. 3
UID	20201128_01	20201210_08	20201208_01

Figure 5:

Explanation	Linescan of Gated Kerr Signal	Theoretical Calculation Kerr Signal
Sample	K3573	Theory
figure	Fig. 5	Fig. 5
UID	20200916_24	KerrMap.txt

Supplementary Figures:

Suppl. Fig.1/2:

Explanation	Polarization dependent Raman	Bias and Gate Dependent KR microscopy
Sample	K3573	K3581
figure	Suppl. Fig. 1	Suppl. Fig. 2
UID	K3573_Raman_polarization.txt	20201206_03

Suppl. Fig.3:

Explanation	Transport Data	Linescan of Kerr Angle	Gate Dependent Spatial Kerr maps with gate voltages -40V.. 40V in 5V steps. (biased over Gr.)
Sample	K3581	K3581	K3581
figure	Suppl. Fig. 3	Suppl. Fig. 3	Suppl. Fig. 3, (plotted: -30V,5V,30V)
UID	20201126_02	20201204_04	201205_spatial_gate_sweep_graphene 201205_110750, 201205_130926, 201205_151059, 201205_171234, 201205_191412, 201205_211542, 201205_231714, 201205_231714, 201206_011849, 201206_032015, 201206_052138, 201206_072307, 201206_092432, 201206_112601, 201206_152911, 201206_173045, 201206_193220, 201206_132737

Suppl. Fig. 4:

Explanation	Transport Data	Linescan of Kerr Angle	Gate Dependent Spatial Kerr maps with gate voltages 30V -30V and 10V. (biased over Gr.)
Sample	K3105	K3105	K3105
figure	Suppl. Fig. 4	Suppl. Fig. 4	Suppl. Fig. 4 (30V, -30V, 10V)
UID	20201218_01	20210107_01	210107_013146, 210107_070612, 210107_032316

Suppl. Fig. 5:

Explanation	Linescan of Power Dependence of Kerr rotation, both lobes can be extracted	Power Dependence of Kerr Signal on left and right lobe of the interface
Sample	K3573	K3581
figure	Suppl. Fig. 5	Suppl. Fig. 5
UID	20200919_02	201211_173227, 201211_174213

Suppl. Fig. 6:

WTe2- Hallbar, measurement of Kerr rotation for different Bias configurations. The bias configuration is indicated in “Explanation”

Explanation	a_axis_long	a_axis_short	b_axis_right	b_axis_left
Sample	Hallbar K3141	Hallbar K3141	Hallbar K3141	Hallbar K3141
figure	Suppl. Fig. 6	Suppl. Fig. 6	Suppl. Fig. 6	Suppl. Fig. 6
UID	210202_094136	210202_160509	210202_112725	210202_203621

Suppl. Fig. 7:

Explanation	Gate Dependent Line Scan of Kerr Signal
Sample	K3573
figure	Suppl. Fig. S7
UID	20200916_24

Suppl. Fig. 8:

Explanation	Polarisation Dependence Gr-WTe2 crossdevice	WTe2 Hallbar
Sample	K3573	K3645
figure	Suppl. Fig. S8	Suppl. Fig. S8
UID	20200921_10	20210322_09
