# Supplementary figures and images for: Berry curvature-induced local spin polarisation in gated graphene/WTe2 heterostructures
Source: Nat Commun. 2022 Jun 7;13:3152. doi: 10.1038/s41467-022-30744-3 (PMC9174237; doi:10.1038/s41467-022-30744-3)

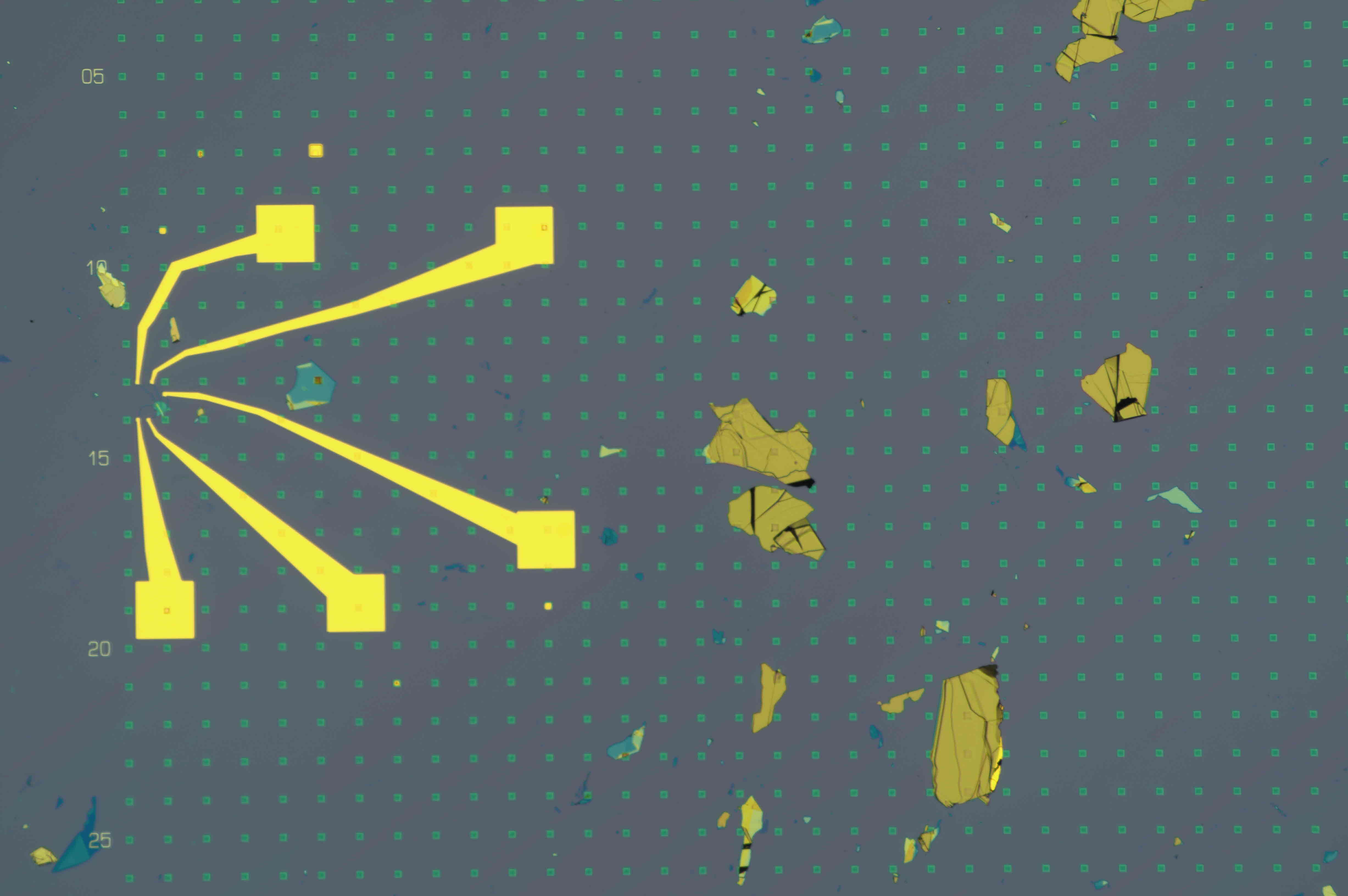

Supplement: Supplementary file 3 — Source Data [file 41467_2022_30744_MOESM3_ESM.zip › WTe2_Graphene/Devices_optical/K3105/K3105.jpg]

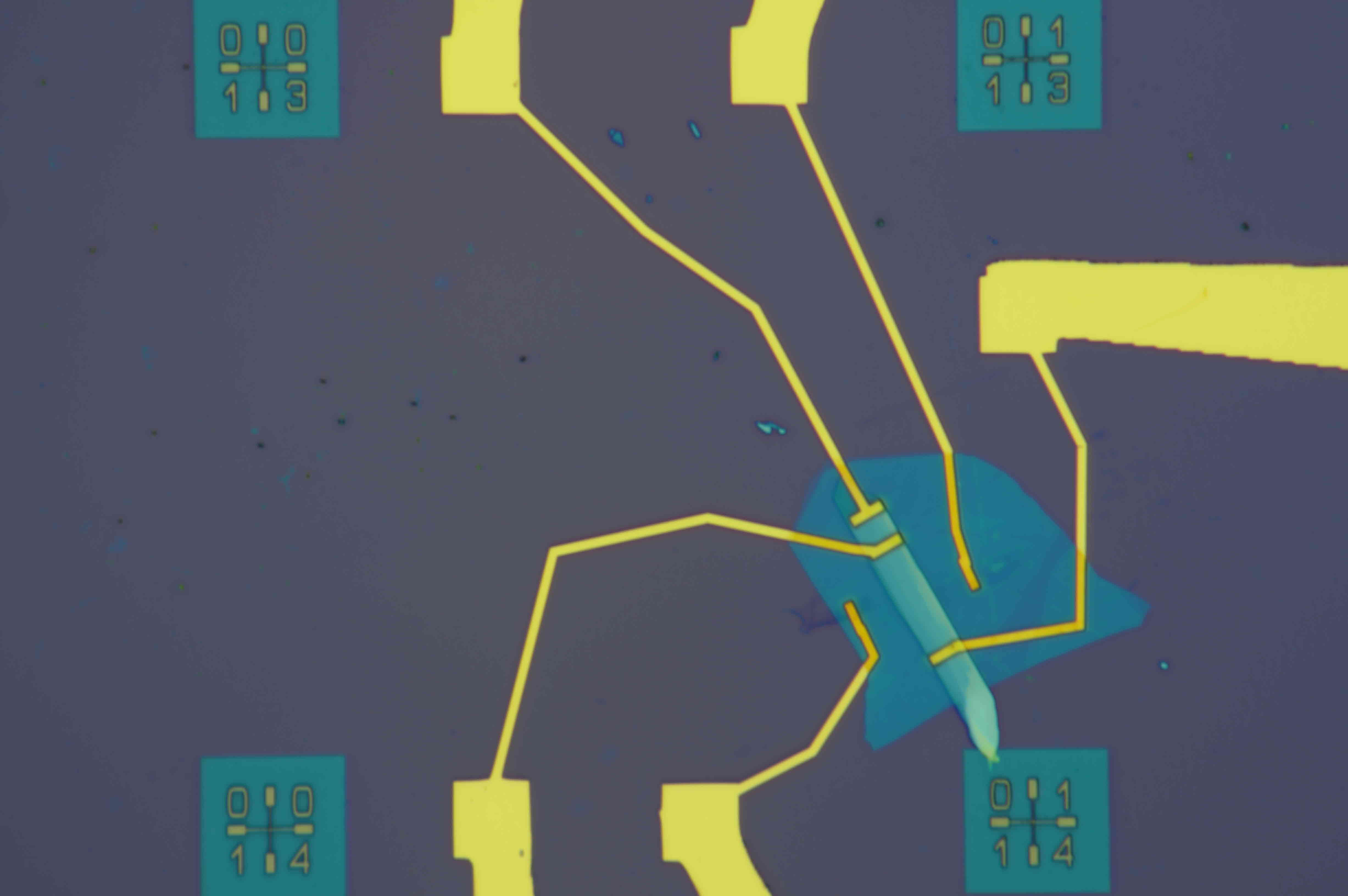

Supplement: Supplementary file 3 — Source Data [file 41467_2022_30744_MOESM3_ESM.zip › WTe2_Graphene/Devices_optical/K3105/K3105_100x.jpg]

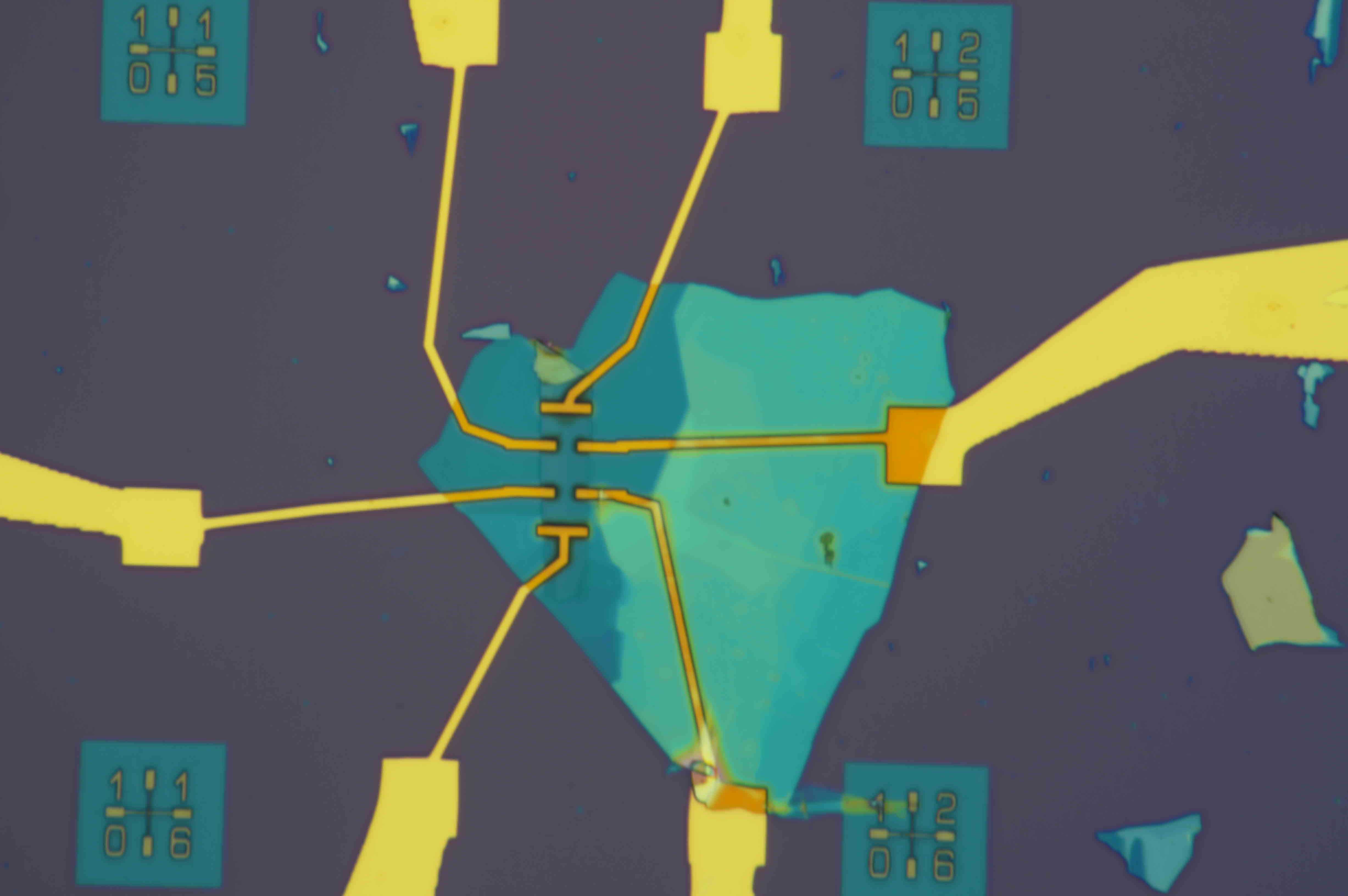

Supplement: Supplementary file 3 — Source Data [file 41467_2022_30744_MOESM3_ESM.zip › WTe2_Graphene/Devices_optical/K3141/3141_100x.jpg]

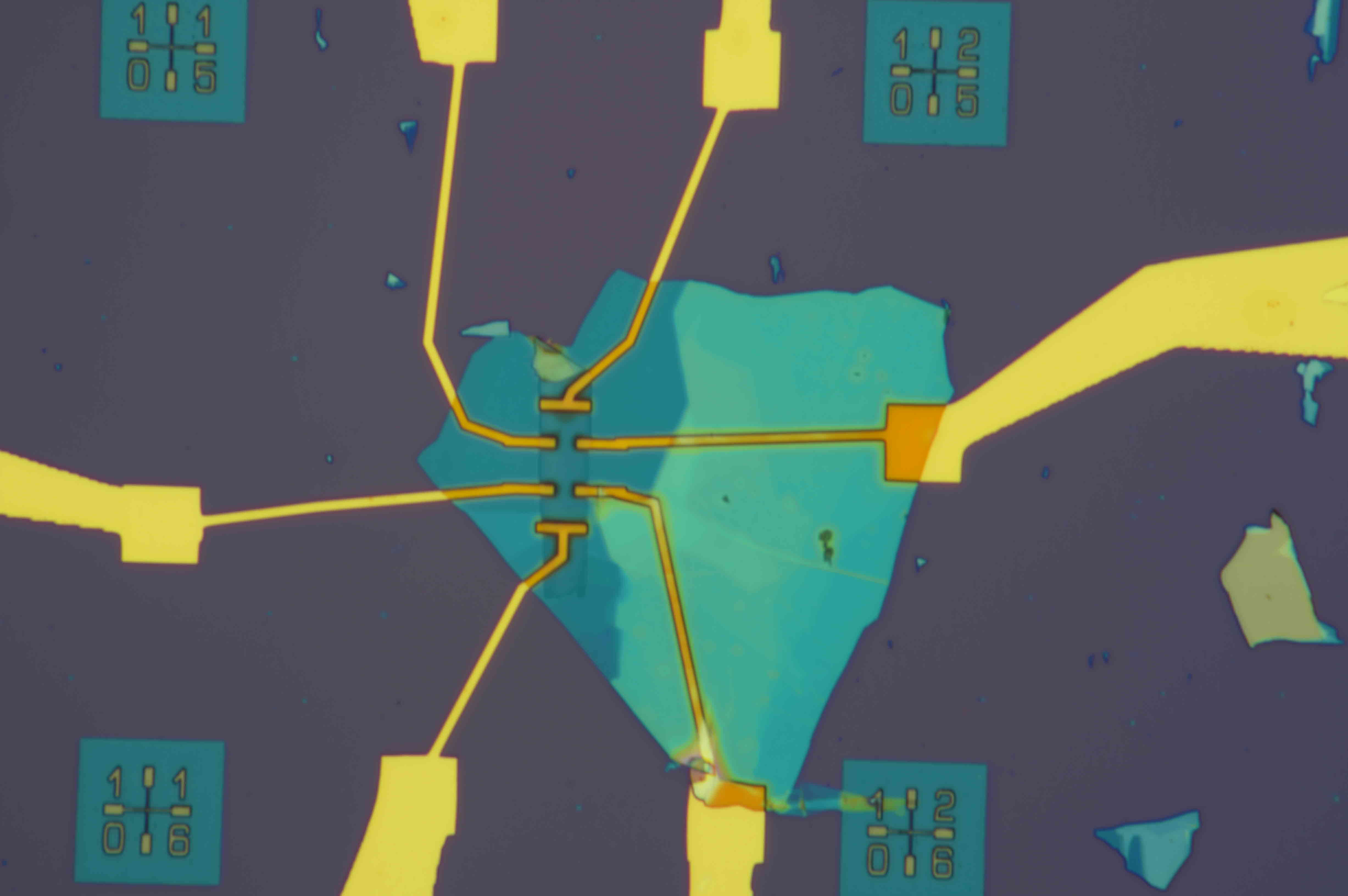

Supplement: Supplementary file 3 — Source Data [file 41467_2022_30744_MOESM3_ESM.zip › WTe2_Graphene/Devices_optical/K3141/3141_100x2.jpg]

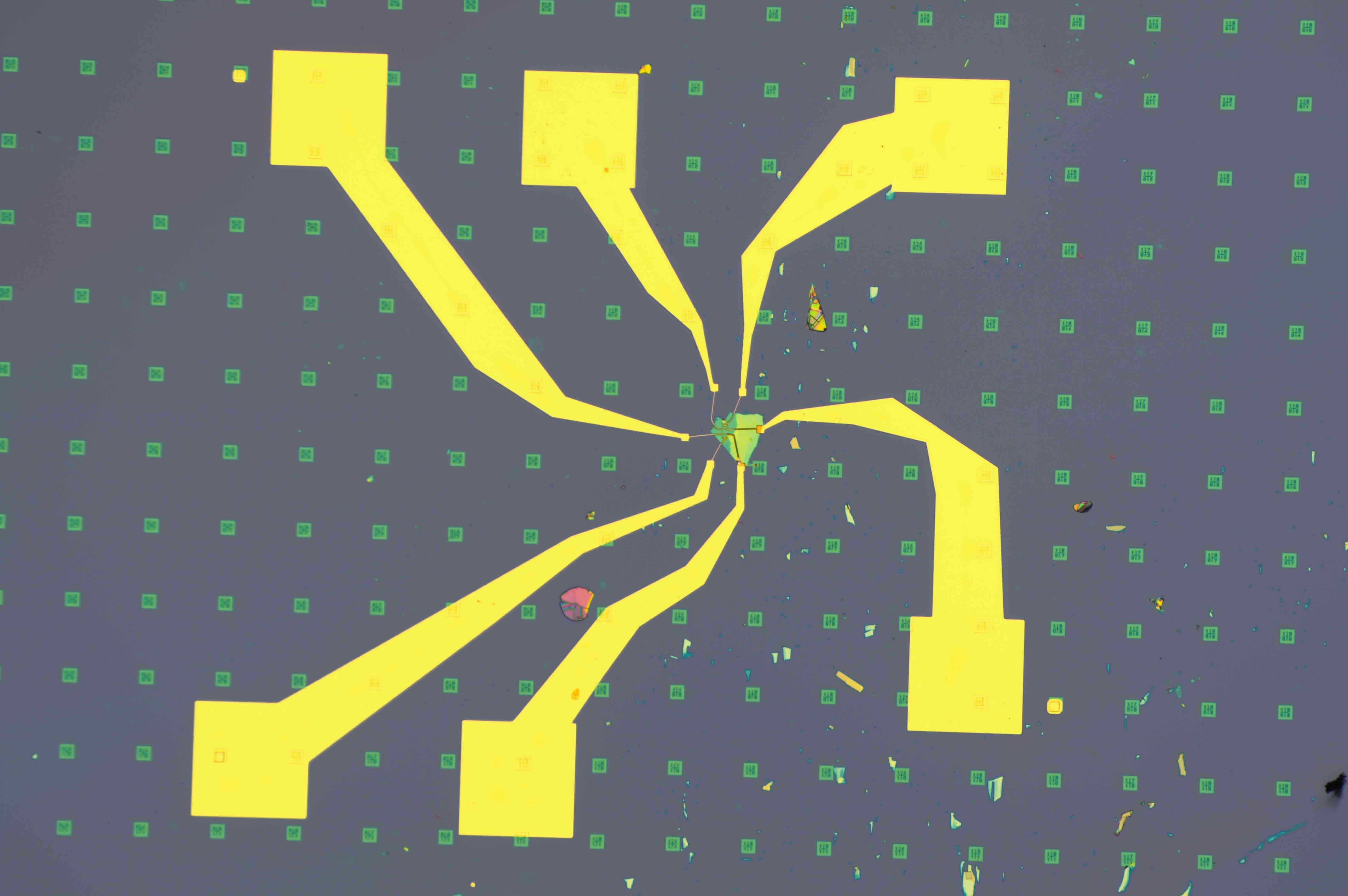

Supplement: Supplementary file 3 — Source Data [file 41467_2022_30744_MOESM3_ESM.zip › WTe2_Graphene/Devices_optical/K3141/3141_10x.jpg]

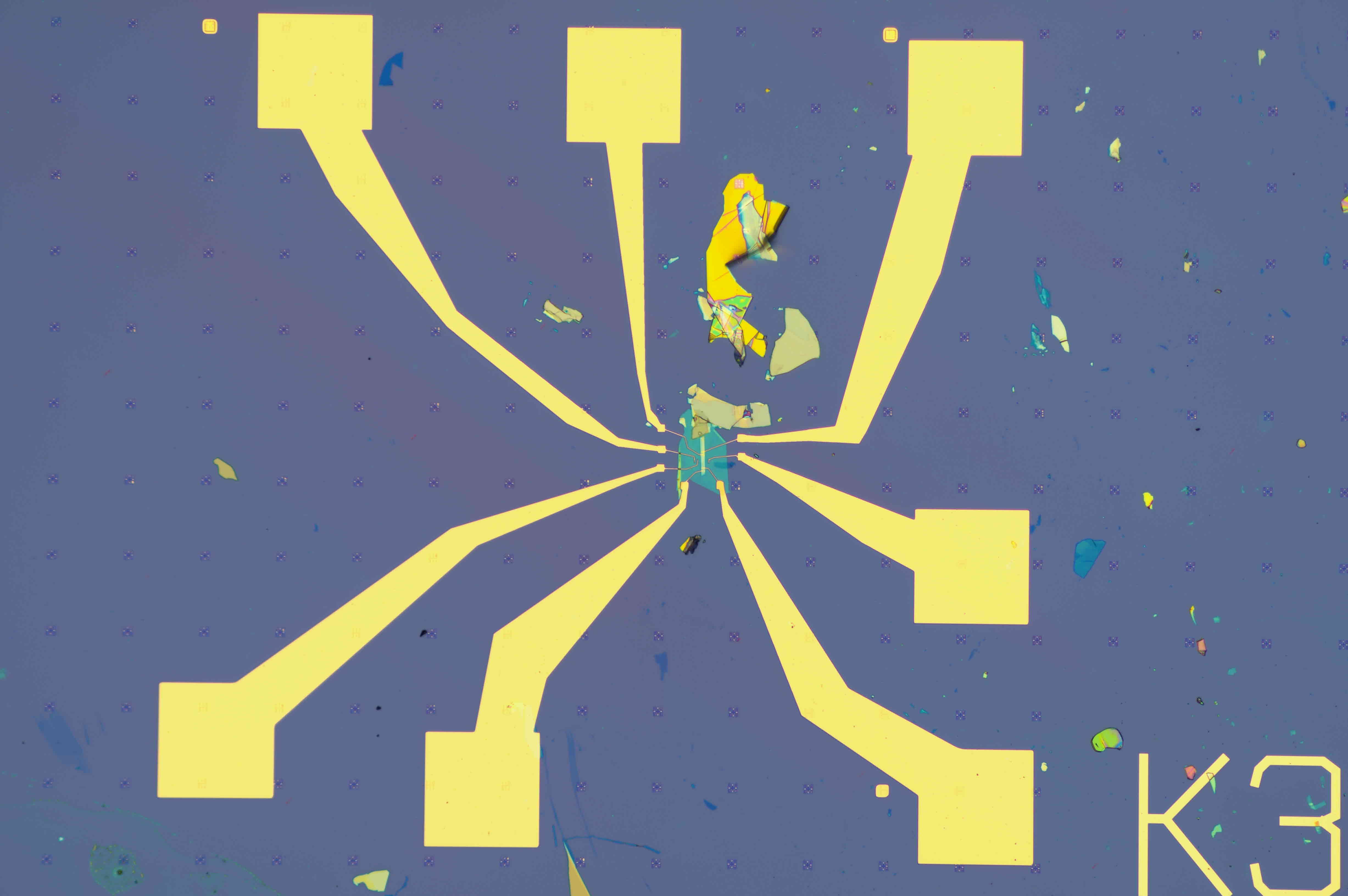

Supplement: Supplementary file 3 — Source Data [file 41467_2022_30744_MOESM3_ESM.zip › WTe2_Graphene/Devices_optical/K3573/K3573.jpg]

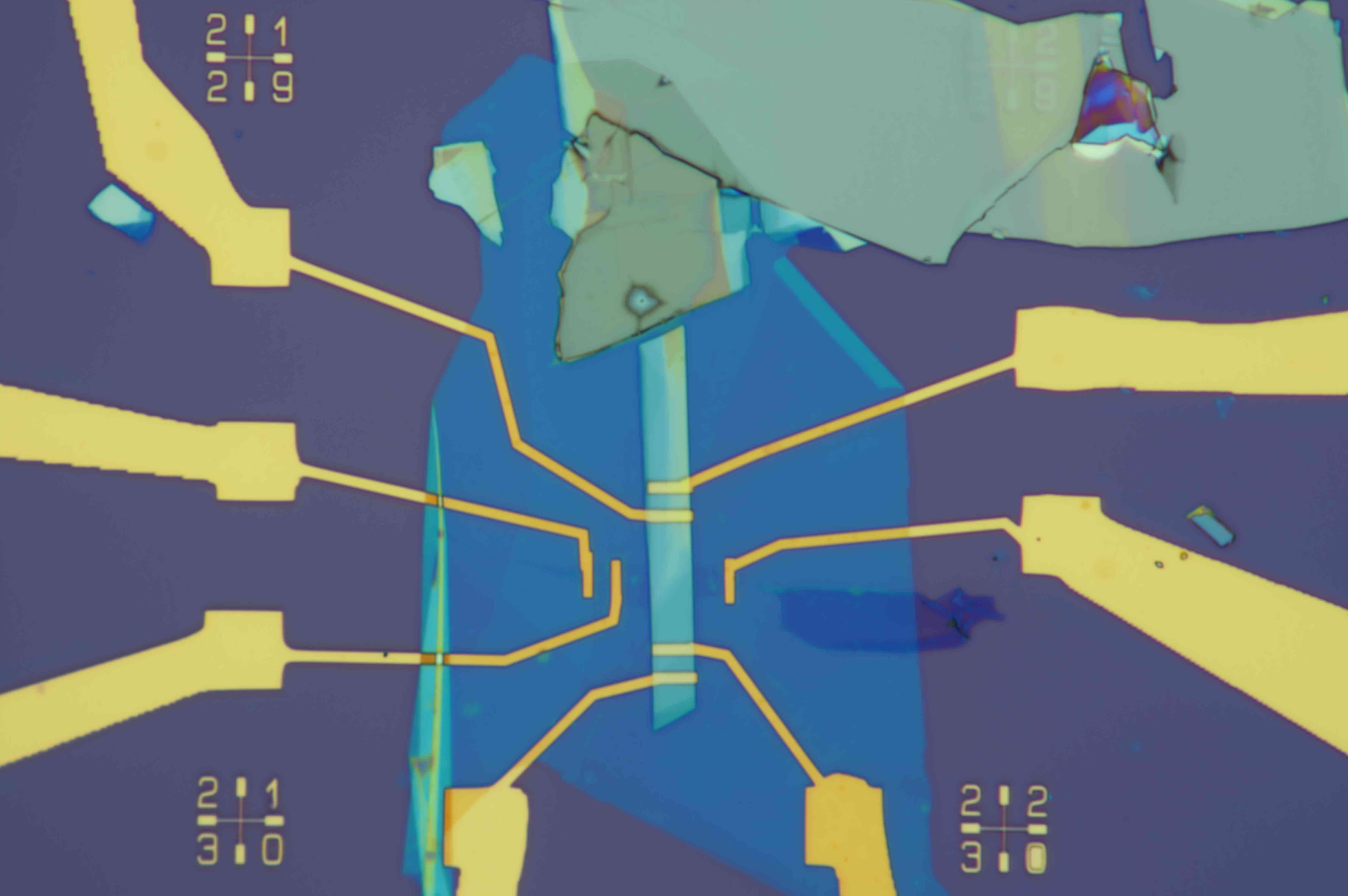

Supplement: Supplementary file 3 — Source Data [file 41467_2022_30744_MOESM3_ESM.zip › WTe2_Graphene/Devices_optical/K3573/K3573_100x.jpg]

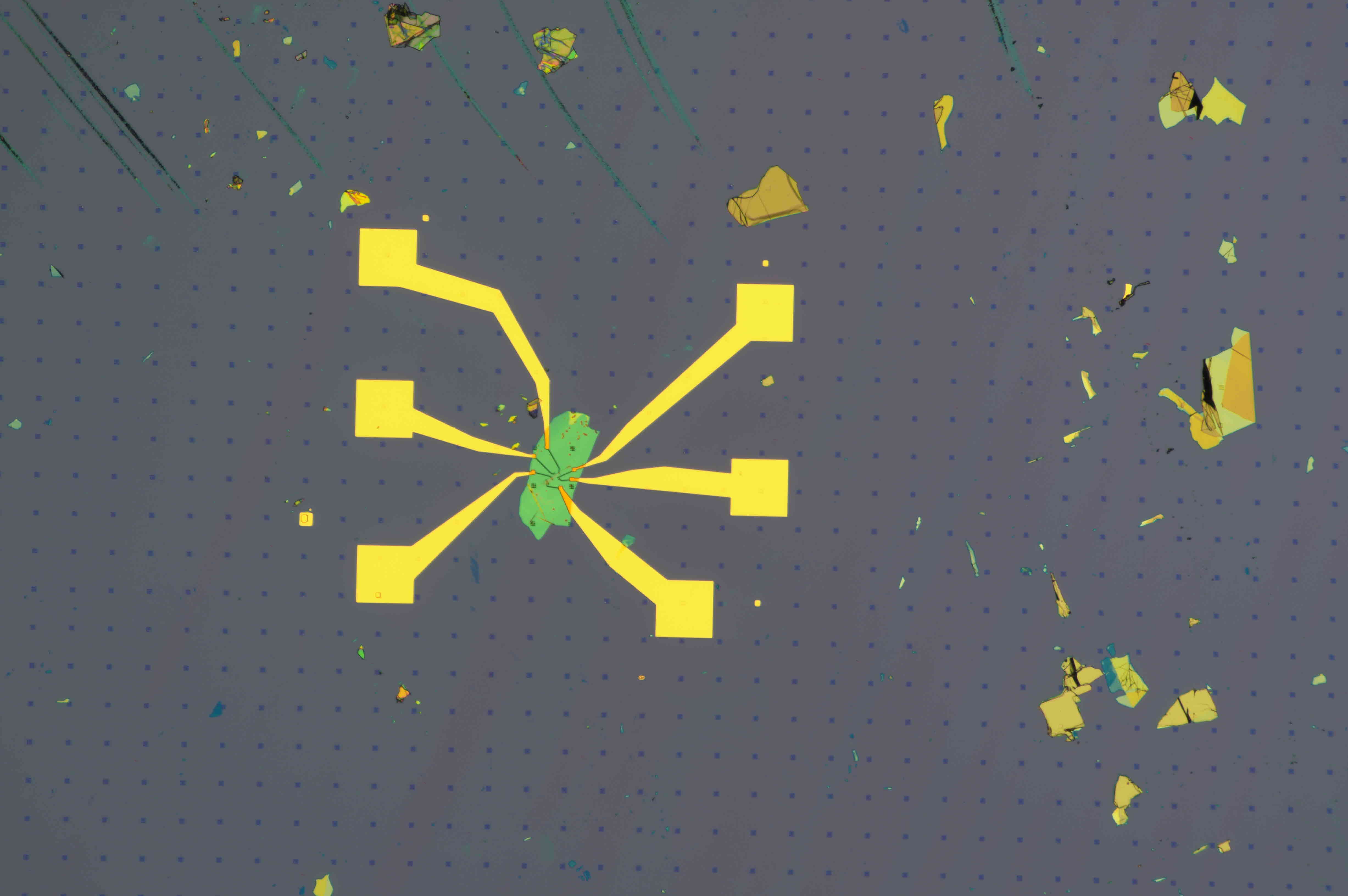

Supplement: Supplementary file 3 — Source Data [file 41467_2022_30744_MOESM3_ESM.zip › WTe2_Graphene/Devices_optical/K3581/1.jpg]

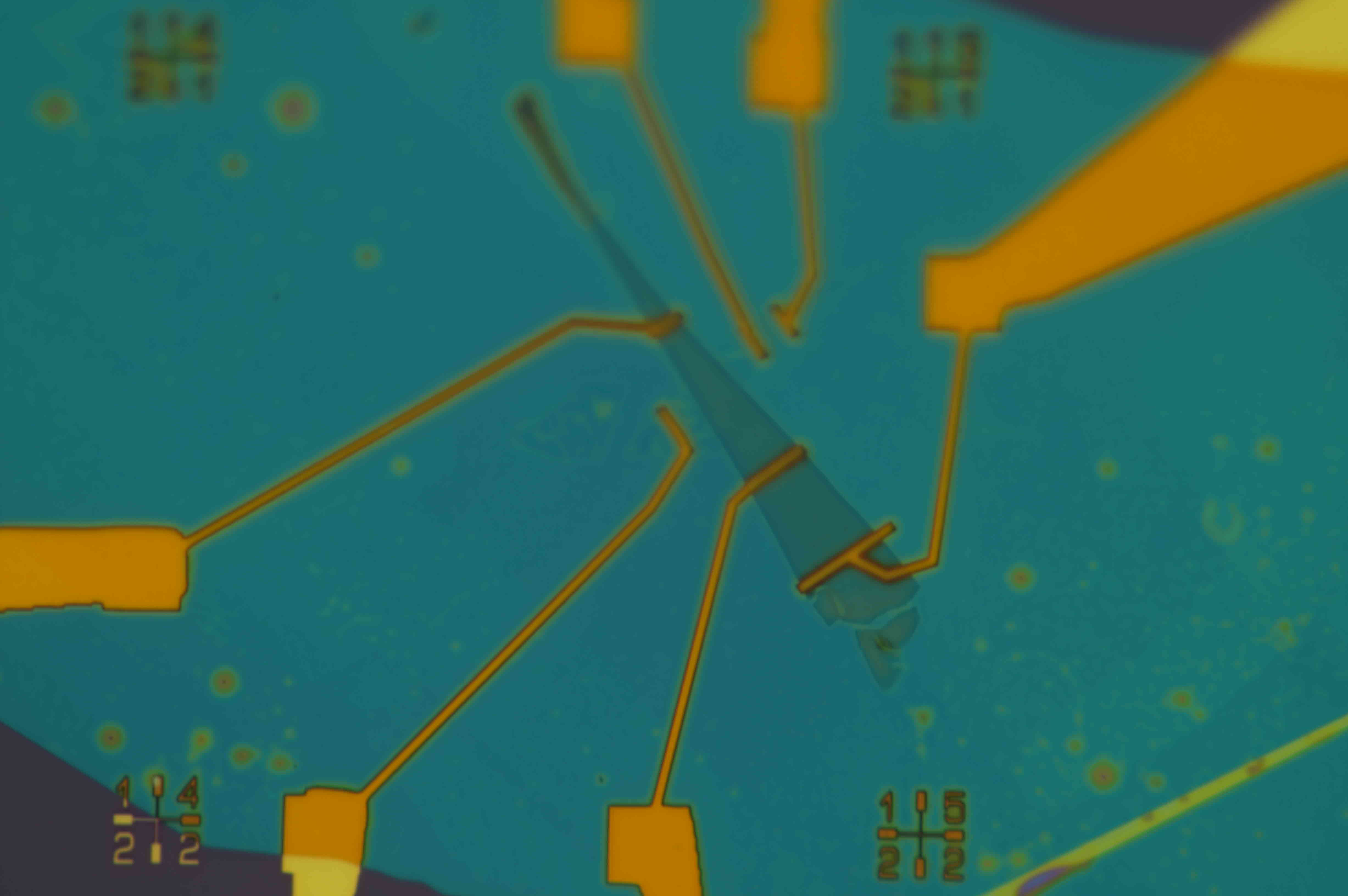

Supplement: Supplementary file 3 — Source Data [file 41467_2022_30744_MOESM3_ESM.zip › WTe2_Graphene/Devices_optical/K3581/3581_100x.jpg]

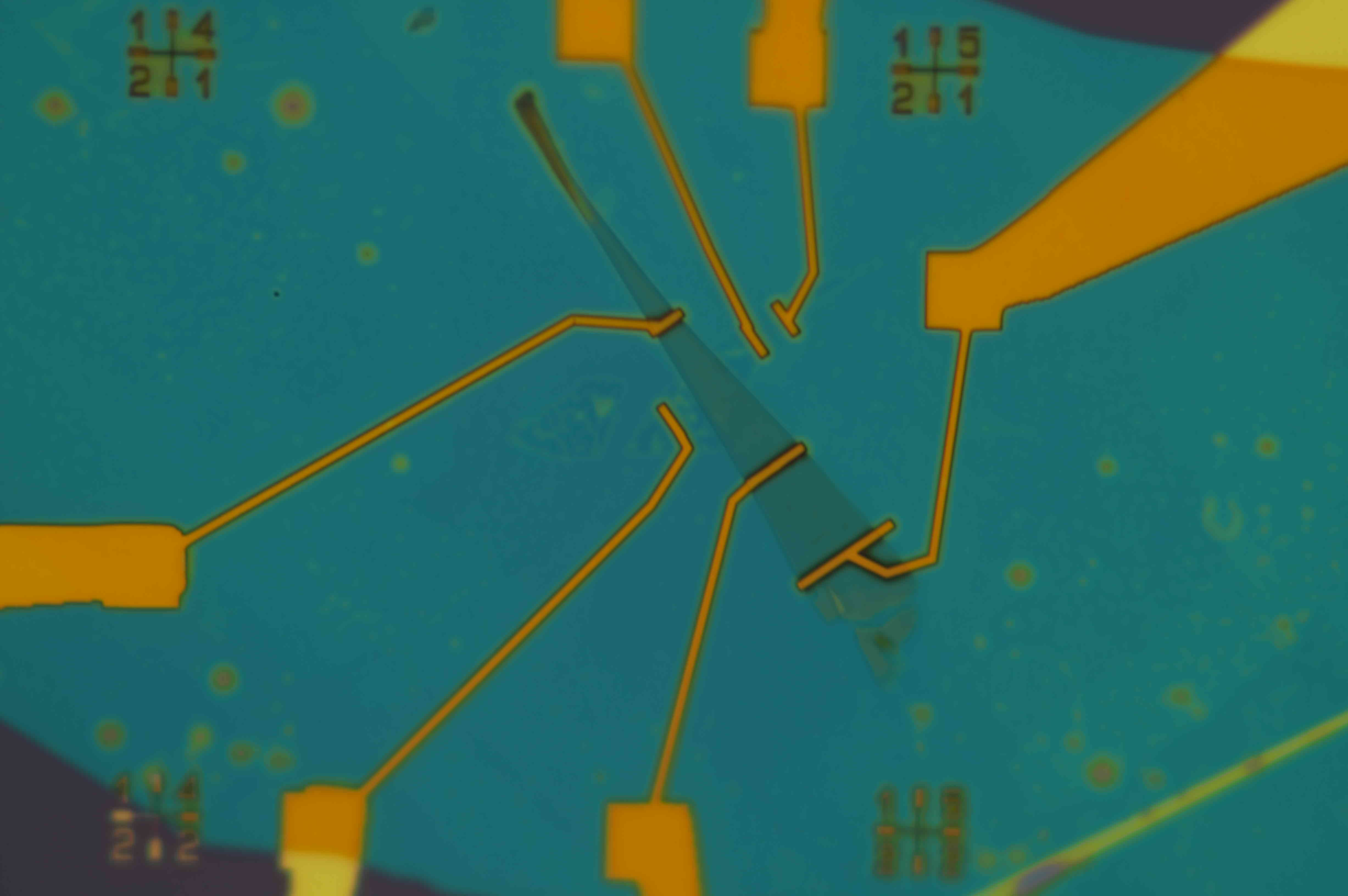

Supplement: Supplementary file 3 — Source Data [file 41467_2022_30744_MOESM3_ESM.zip › WTe2_Graphene/Devices_optical/K3581/3581_100x_2.jpg]

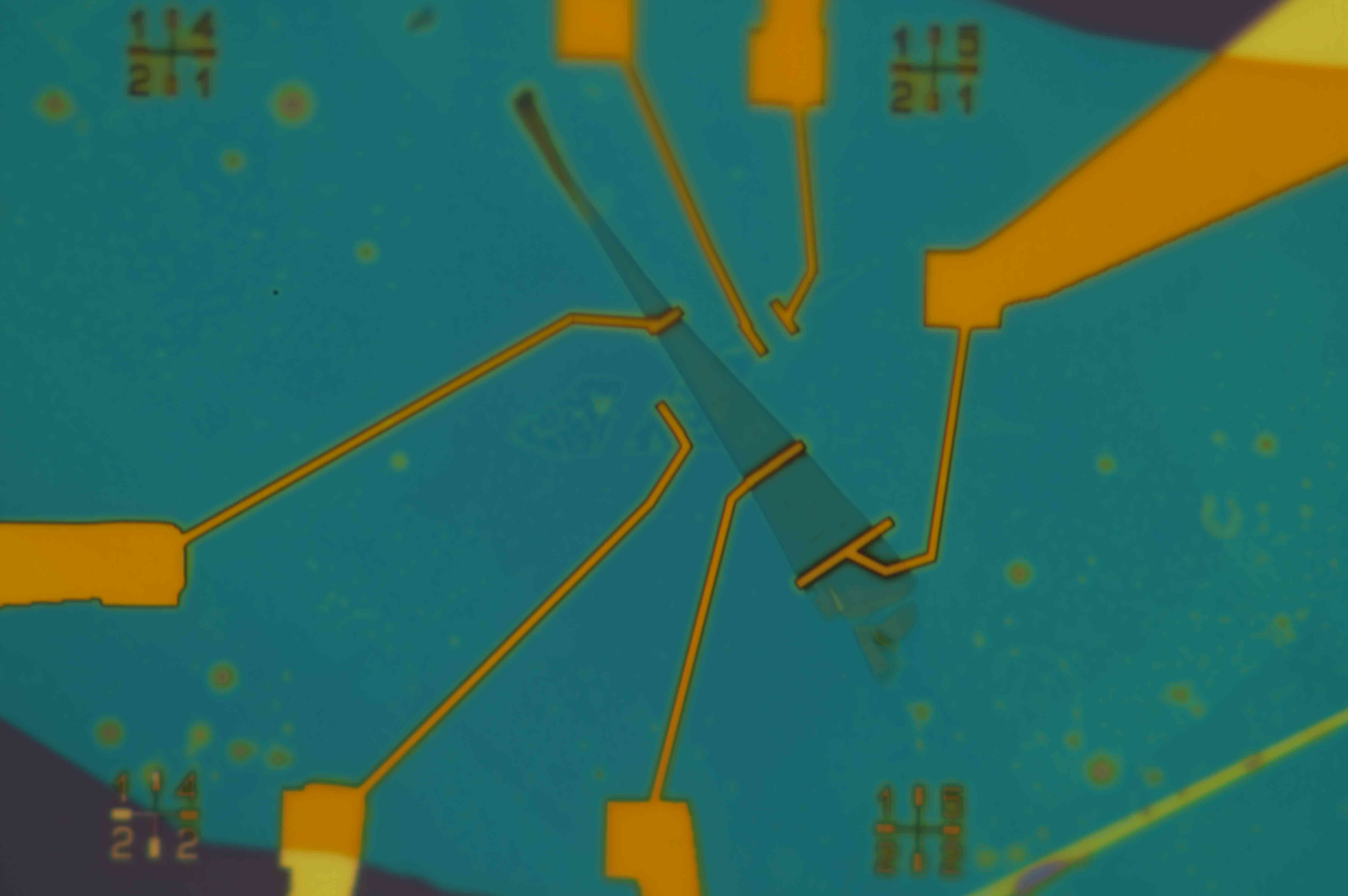

Supplement: Supplementary file 3 — Source Data [file 41467_2022_30744_MOESM3_ESM.zip › WTe2_Graphene/Devices_optical/K3581/3581_100x_3.jpg]

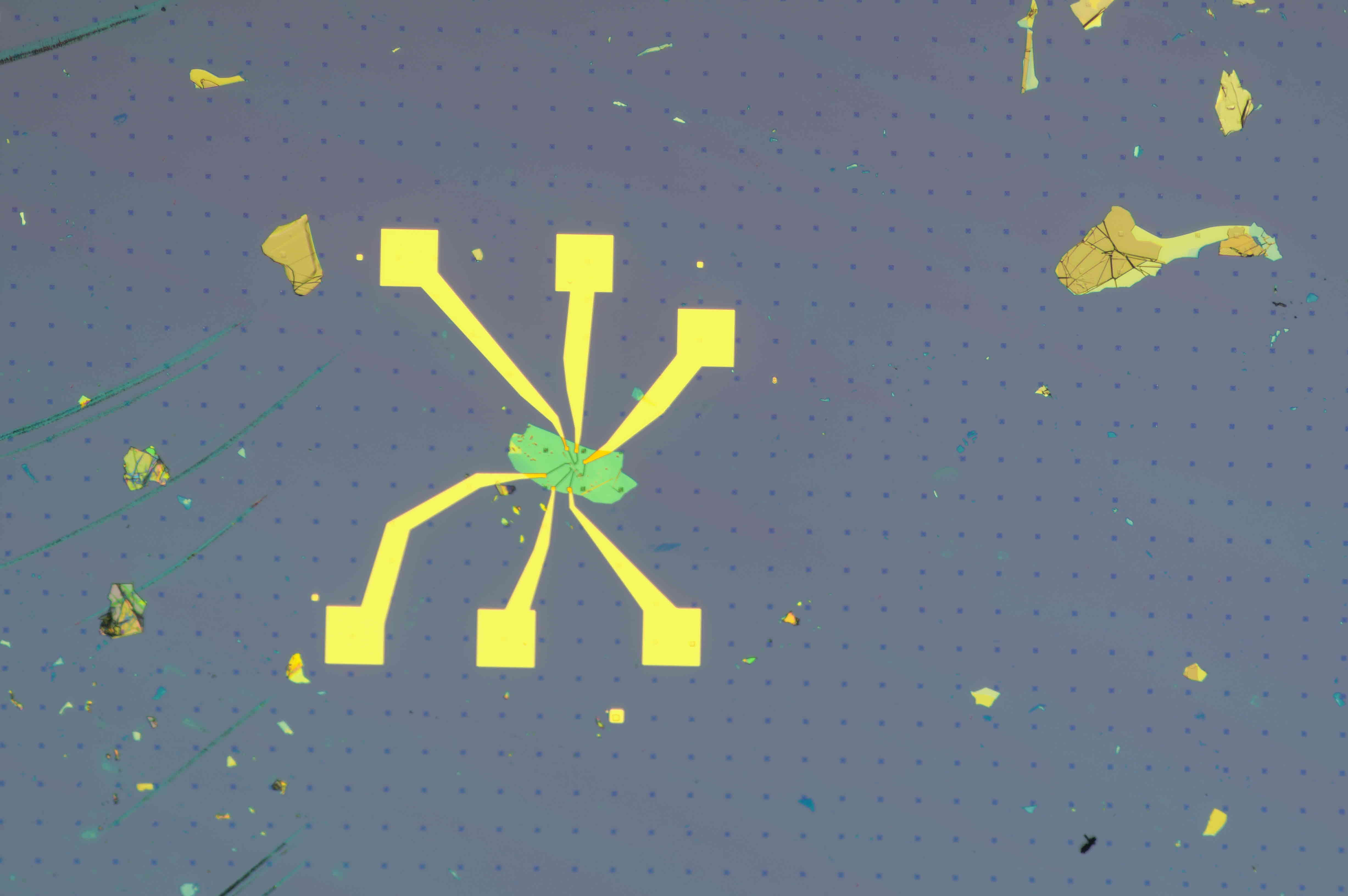

Supplement: Supplementary file 3 — Source Data [file 41467_2022_30744_MOESM3_ESM.zip › WTe2_Graphene/Devices_optical/K3581/3581_10x.jpg]
